# Medical Evidence in Support of the Commission

**Published:** 1852-04-01

**Authors:** 


### MEDICAL EVIDENCE IN SUPPORT OF THE COMMISSION.

*Mr. George Cornelius Johnson*, examined by Sir F. THESIGER.—I am a general practitioner. I have known Mr. Ince for thirty-four years. In the latter part of the year 1845, and the early part of 1846, I attended Captain Cumming in Belgrave-terrace,—that is, from the 10th April to the 30th April, 1845; also in the months of May and June, and in April and May, 1846. Captain Cumming was in a state of great bodily weakness at that time. He was suffering from low fever—that was the specific complaint for which I attended him. — Q. Had he other complaints. A. He had a disease of the prostate gland, and as the result of that he had considerable difficulty in passing his water at times. — Q. Independently of his advanced age, would you not consider that as quite sufficient to prevent the possibility of any sexual intercourse? A. I do not hesitate to say that it would be quite impossible for him to have sexual intercourse. — Q. What was the conduct of Mrs. Cumming towards Captain Cumming in general, as far as your observation went. A. It varied very much. At times she was kind and attentive to him, and apparently anxious about him—anxious that I should do everything which my art enabled me to do to restore him to health. At other times it would be very violent, and her language would be violent—she would address him in an angry tone, and would exhibit great indifference as to the result of his illness. — Q. You say that she would be very violent at times towards him—would she in your presence exhibit that violence? A. In language and occasionally in gesture; it was never carried beyond that. There was never any personal violence. — Q. Are you able to say whether she attended to his wants, and supplied him with those things which he required? A. There were times, and they were very frequent indeed, that I had to complain of the want of attention. You must understand that at this time he was in a perfectly helpless condition; he was confined to his bed. — Q. Was that during the whole of your attendance upon him? A. No—during the early part of it. He was prostrate with fever.—Q. And was it during this period that she exhibited this variety of conduct towards him? A. Precisely.—Could you observe whether Captain Cumming had given her any particular provocation? A. Not in my presence. — Q. Did she ever speak to you anything about her husband? A. I am afraid at this distance of time I cannot charge my memory with that sufficiently to state on oath. I am anxious to be very careful and very correct. — Q. Did she state any other matters to you at any time that excited your attention? A. Oh yes! She stated frequently, in reference to her daughters, and also more especially towards her daughter Mrs. Ince, and also against her son-in-law. I made an observation to Mrs. Cumming that I thought it strange that another medical man should have been called in to the family when she had a son-in-law, an experienced man in the profession. That observation I repeated from time to time, and it always originated the same remarks from her—that the reason she did not employ her son-in-law was, that he was a thief, and that he, in fact, would lay his hands upon anything. On my remonstrating with her upon my feeling that it was impossible that a man in his position and character should be guilty of anything of the kind, she said, "Oh, yes; he has done so, and I can give you an instance; he has stolen from me a silver bread-basket or basket." — Q. You say that Mrs. Cumming stated this upon several occasions with regard to Mr. Ince? A. Several occasions. — Q. And you endeavoured to dissuade her from entertaining any such notion. A. I did so. — Q. And could you prevail upon her? A. No, indeed I could not. — Q. What did she say about Mrs. Ince? A. She spoke of her as a person totally unworthy of her affection. — Q. Did she give you any instances—did she say in what respect she was unworthy of her affections? A. That she had been unkind to her. I can hardly again charge my memory with any special observations which she made in reference to that matter. I have heard a greal deal, but of course that does not come under my cognizance. — Q. Did she appear to have any antipathy to her daughters? A. She seemed to have a perversion of all natural feeling, I thought, towards her children. — Q. Did she speak to you about Mrs. Hooper? A. Very likely—I do not remember that she did particularly—the observations were general; but there were instances in which she particularly alluded to Mrs. Ince.— Q. Had you any opportunity of seeing what state Mrs. Cumming's room was in? A. No; I was never admitted into that room, I think, but upon one occasion.—Q. What was your opinion, from the opportunity you had of seeing Mrs. Cumming, as to her state of mind? A. That she was of unsound mind.

By the Commissioner.—When did you first come to that conclusion? A. Certainly within the first week of my attendance.

By Sir F. Thesiger.—And as your acquaintance with Mrs. Cumming advanced, and your opportunities of observing her continued, did you come to that conclusion satisfactorily in your own mind? A. My first impressions remained entirely unaltered. — Q. I believe that you signed the certificate by which she was confined in York House? A. I did. (The certificate runs as follows:—"I, George Cornelius Johnson, being an apothecary legally qualified, hereby certify that I have this day, separately from any other practitioner, visited and personally examined Mrs. Catherine Cumming, the person named in the accompanying statement and order; and that the said Catherine Cumming is a person of unsound mind, and a proper person to be confined; and that I have formed my opinion from the following facts:—viz. delusions as to the character of her near relations; delusion as to being robbed by them and others; violent conduct towards her husband; and unfounded antipathies towards her children.") — Q. Upon your signing this certificate, did you examine Mrs. Cumming, and find that these delusions were operating upon her mind? A. Yes. — Q. Have you seen her since that time? A. I saw her during the late commission at the Horns Tavern, and have never seen her since until the present investigation. — Q. Probably you will be able, from your experience, to tell us whether a person of an advanced period of life whose mind labours under delusions of this kind is likely to be restored, or so likely as a younger person? A. Not within my experience.

Cross-examined by Mr. Serjeant Wilkins.—Q. Have you had much experience in diseases of the mind? — A. I have had the average which usually falls to men in general practice. I have not had any great experience in matters of this kind. It is quite due to you that I should admit that. — Q. During the times that you visited Captain Cumming, had you frequent conversations with Mrs. Cumming? A. Yes, I had. — Q. Long conversations? A. Not long. — Q. Were you aware at that time that she was addicted to habits of intemperance? A. I never saw her so. — Q. You have stated that her conduct varied very much. Supposing that to be so; that she was in the habit of indulging in strong drinks, would you be at all surprised to find that the case? A. Certainly not; both states would lead to the same results. — Q. You have stated that in your opinion Captain Cumming would be incapable of sexual intercourse? A. That is my opinion. — Q. First of all, let me ask you, although there might be incapability of intercourse, is there any impossibility that the desire should exist? A. No, I should say not. — Q. What induces you to suppose that the sexual intercourse was impossible? A. The disease of the prostate gland, and the organs of generation generally—I should say it would be physically impossible. — Q. You say as to the organs of generation generally. Was there any other indication of disease but that of the prostate gland? A. It did not fall within my observation. — Q. Did you notice at times when you visited Mrs. Cumming, that she appeared more excited than at other times? A. Yes. — Q. Can you tell me as a matter of fact, from your own knowledge of the operations of the mind, is not the mind, generally speaking, much more prone to brood over past wrongs under the influence of drink than at other times? A. I am not quite clear that your position is right. On the contrary, I think rather differently.—Q. Do you remember a case in which a man lately destroyed a child? A. Yes, you may cite isolated cases; but I think as a principle it is not the fact. — Q. But there are isolated cases? Was it not when she was in that excited state that she spoke of the family in the way you have described? A. No. — Q. You have assumed that all those impressions on her mind were delusions? A. I did so at the time, and I think so still.—Q. And under that impression it was that you came to the conclusion that she was insane? A. It was so. — Q. An insane person may argue shrewdly on non-existing facts, may he not? A. Yes. — Q. And it is the non-existence of the premise that induces the belief of unsoundness of mind, there being no real cause? A. No real cause. — Q. You would not call imperfect reasoning a proof of insanity? A. Imperfect reasoning? Do you mean in points submitted for general argument? — Q. No. Supposing, for instance, that there may have been some very trifling circumstance which may have existed upon which one may attach very important consequences, would you argue that as a proof of insanity? A. Well, generally I might not, but individually I might. — Q. That means nothing. A. That is just what I wish it to mean; because it is a position which will not admit of explanation. — Q. For instance, supposing that I have been wronged by some person, and I am, as many people are, much more prone to suspicion than others—and suppose I afterwards bring my mind to believe that that person would wrong me in much greater particulars, would you say that that was a proof of insanity? A. On the conviction that that party, to my own knowledge, or as far as I had an opportunity of forming an opinion, had not done any injury. — Q. You must take my entire hypothesis. I ask you this—supposing some person has done me a slight injury, would you say, because I afterwards suspect that person of greater wrong that therefore I am insane? A. Certainly not.—Q. You talked about perversion of natural feeling, and not antipathy under consciousness of wrong—unnatural feeling? A. Yes. — Q. And may not that antipathy, even in the case of mother and child, proceed to estrangement and dislike? A. It is quite possible, but not to the expression of such opinions as Mrs. Cumming gave utterance to. — Q. You say there was a perversion of all natural feeling towards her children—in what way did she show her antipathy to Mrs. Hooper? A. I have already explained that; I cannot charge my memory with any particular explanation with respect to Mrs. Hooper, but she generalized them as her children, and spoke of their cruelly unkind conduct to her. — Q. Will you be kind enough to furnish to the jury your definition of unsoundness of mind? A. It is a very extensive question.— Q. I wish to have a definition, if you please. A. My impression is, that unsoundness of mind involves acts which are naturally contrary to reason, and altogether at variance with the circumstances and the condition of life of the party—peculiarity of habit, manner, and conversation inconsistent with the ordinary circumstances of life.—Q. Now will you be kind enough to tell me what it was that induced you to believe beyond what you have stated in your certificate, if there be anything, that Mrs. Cumming was of unsound mind? A. I should first mention the fact of my impression concerning the delusion of her children having robbed her—that appeared to me to be inconsistent—the peculiarity of her own person, manner, and habits. — Q. Will you state what that peculiarity was? A. In the first place, I should tell you my visits were made at different times of the day—that she was always wrapped in a loose dressing-gown, and otherwise in appearance very different from what one would have expected from a person in her position in life. Again, the general appearance of the house showing either that she had no power, or having the power, did not choose to exercise it, in the arrangements of the house—the wretched condition of the room in which my poor unfortunate patient was placed, totally inconsistent either with the character of such a case of illness, and without any of the comforts one would naturally have expected to have found in the house of a person having, as I presumed, the fortune of Mrs. Cumming. — Q. Are those your entire reasons? A. The generally offensive character of the house. — Q. Did you know that she was complaining of illness? A. She never did to me; she once consulted me, but that was about some affection of the eyes. — Q. Do you know that she was at that time attended by some medical man herself? A. I am not aware of it. — Q. You have spoken of the mean way in which her house was furnished—were you aware that her furniture had some time before been seized to pay a gambling debt of her husband's? — A. No. Had I been aware of that, it would have been an excuse to me that the house was in a bad state. — Q. Did you know that after her marriage with Captain Cumming he had children affiliated to him? A. No, I did not: it may be as well to state to you that I do not know that I had even heard the name of this family until I had been called in.

Re-examined by Sir F. THESIGER. — Q. You have been asked as to certain general principles as to unsoundness of mind. Is it your judgment that those general principles can be applied to my case, or must each particular case be judged of by its own circumstances? A. I should say upon its own merits.

By the COMMISSIONER. — Q. You saw this lady once in her own bed-room? A. Only once; it was during my attendance. I do not know whether you would call it a bed-room; it was a wretched place, and the odour was so insufferable that I was very glad to escape from the short interview I had with her.

A JURYMAN.—Her bed was in that room? A. I cannot charge my memory with that. — Q. You did not go over the rest of the house? A. I never went over it.

*Mr. Thomas Wilmot*, examined by Mr. PETERSDORFF.—I am a surgeon, residing in Chester-street. I have been about fourteen years in practice. I first saw Mrs. Cumming in York House Asylum, on the 14th May, 1846. For the first ten minutes I saw her with Dr. Millengen, the owner of the house; he then left us. I asked her how it was she was there? She entered into the subject, and complained very inveterately of her husband and children. I thought that, under existing circumstances, only natural. — Q. I think it would be desirable that you should give us the conversation as nearly as you can recollect, what you said to her, and she to you? A. I asked her the age of her husband. She had spoken in most disrespectful terms about her husband, and then raved on with violence, which, as I did not think it extraordinary, I did not pay much attention to. She told me her husband was an old man, 79 or 80, I forget which. I asked her by what particular facts she had to complain of her husband's conduct? She answered me that he was a whoremonger, and that he had connexion with every nurse who came near him. She rather dwelt upon this conversation. She stated to me that she had caught her husband lately in the fact. I tried to disabuse her mind, having learned from her that he had disease of the generative organs. I questioned her about her daughters. She said that Mrs. Ince was a prostitute, but did not assign any reason on my asking her. She stated that Mr. Ince had murdered, I think, three children. I am positive to two—two of his own, and one nephew or niece. One of his own, she said, was done for—murdered by him; and subsequently after its death "glazed" over. She told me that Mr. Dangerfield had robbed her of plate and property to the amount of 300*l*. She could not, or would not, afford me any explanation of it. She referred me to Mr. Johnson as her medical attendant, who would speak to her sanity. That is all I remember. The interview lasted about an hour and twenty minutes. I think she said Mr. Cumming was a confirmed drunkard. I considered that she was labouring under delusions as to most of those points; that she was a person incapable of taking care of herself; that she was unsound or incapable of taking care of her property. She said her house was in perfect order. I went immediately to the house in Belgrave-terrace. I saw Captain Cumming. He was in the front parlour. His room was scantily furnished; the bed and everything was very unpleasant. His disease would tend to make it unpleasant. I found him a person totally incapable, in my opinion, of having any desire for the other sex. The poor man was some three minutes before he could pass off a teaspoonful of water. I went over the other part of the house. I found it in perfect disorder. The upper rooms were devoted to the pigeons, the lower ones to the cats. I afterwards attended Captain Cumming, and Mr. Ince saw him occasionally. He died on the 10th of July. I never saw Mrs. Cumming again till she was at the Horns Tavern. I had no conversation with her then. I signed the certificate after seeing the house.

Cross-examined.—I did not sign the certificate until after she was removed to the asylum. At that time Mr. Ince had attended patients for me in my absence, and we were on a friendly footing. I think Mr. Ince knew nothing of Dr. Millengen at that time. — Q. When you first went, did you not find her in a great passion? A. Perhaps I put her in a great passion. — Q. When you say she said that Mrs. Ince was a prostitute, was the word "whore," or "prostitute," used? A. "Prostitute:" she used the word "whore," over and over again, in connexion with her husband and Mrs. Ince both. — Q. This was at the time when she was in this state of passion? A. Oh, no.— Q. Was she cool then? A. Oh, she did so, but I did not form my opinion on anything she said until she had quieted herself. I considered that she had been removed from home, and that it was likely that she would be excited. — Q. When she complained of the disease, I believe you say she stated that the captain had disease in the generative organs? A. Those were not her words; but she said that in effect. — Q. Did she attribute this to his early excesses? A. No.—I have seen children who have died of scarlatina. It is not in my experience the case that, generally speaking, after death they present a sort of glazed appearance of the skin. — Q. Will you be kind enough, if you please, to give us your definition of insanity? A. Upon my word I have never seen or heard any definition of insanity that was satisfactory to me. — Q. But are there no general rules? A. None that are satisfactory to me. — Q. Then what induced you to pronounce Mrs. Cumming unsound? A. I have already given you my reasons. — Q. You have given us a conversation; I want to know what your reasons are? A. I considered she was labouring under delusions. — Q. Did you ever take the trouble to ascertain whether they were delusions or not. A. I took the trouble of going to the house, and having a conversation with Captain Cumming, before signing the certificate. — Q. To anybody else? A. To no one else. — Q. Did you expect Captain Cumming, if he had been inconstant, would have confessed it to you? A. I did not ask him if he was inconstant—it was absurd. I had seen the man try to make water. — Q. Then you think, do you (reflect for a moment), because a man might have difficulty, extending even to agony, in making water, that would forbid the existence of sexual desire? A. I think the difficulty he experienced would—he might have it mentally, but not in the generative organs. — Q. Desire in the organs; I never heard of the affections being placed in those organs before. I ask you whether the affection of desire itself might not exist. A. I do not hear what you say, (The question was repeated.) A. I do not now understand; if you mean that he had the desire, I do not believe that it did or could exist. — Q. Do you not know, as a medical man, that one of the greatest curses attendant on that disease (stricture, disease of the bladder) is, that desire will exist, and beget the greatest agony? A. My experience is the contrary. I had never more than one interview with Mrs. Cumming. I have not, perhaps, had above six or seven cases of lunacy under my own treatment: under the treatment of others, forty or fifty. — Q. Have you signed certificates for some six or seven? A. Yes. — Q. Upon each of those occasions did you sign certificates upon one interview? A. Oh, no. — Q. Was this the only case in which you signed a certificate on one interview? A. I cannot say that. I may have seen the patient only once. I never signed one without the evidence convinced me I ought to sign it. — Q. Your recollection is that, except in this case, you never signed a certificate on one interview? A. If I had not done so before, I have since.

A Juryman.—Have you seen Mr. Ince this morning? A. I have. — Q. In what state is he? A. He is in nearly the same he was on Saturday. He is threatened with paralysis.

Mr. Serjeant Wilkins.—Will you allow me to ask what are the symptoms which you say threaten paralysis? A. Amaurosis: he has partially lost the sight of one eye; he has numbness in his hand; and had giddiness in getting out of his carriage a day or two ago, before he consulted me.

At this stage of the proceedings, the Commissioner, addressing Sir F. Thesiger, asked him if he considered the examination of the lady as a part of his case.

Sir F. Thesiger.—I have always understood that those who support the commission should produce the lady.

The COMMISSIONER.—The usual course is, that the lady is examined at the end of the plaintiff's case, and again at the end of the defendant's case; and when the jury think fit.

Mr. Serjeant WILKINS proceeded to examine the witness as to the number of certificates in lunacy he had signed. He considered thirty was the limit.

*Mr. William Bloxam* was called. Is the present medical officer of York House Asylum, Battersea: knew Dr. Millengen, the former proprietor, who is now dead. The witness produced the book kept by Dr. Millengen during the time Mrs. Cumming was an inmate in that asylum.

Sir F. THESIGER proposed to offer the entries made by Dr. Millengen of Mrs. Cumming's case as evidence. Mr. Serjeant Wilkins objected: and a long discussion ensued. Sir F. Thesiger relied on the 60th sect. of the 8th and 9th Vict., c. 110, of the "Lunacy Act," which directs that "there shall be kept in every licensed house, &c., a book, to be called a case book, in which the physician, &c. shall make entries of the mental state of each patient, together with a correct description of the (treatment) of his disorder," &c. He contended that the book so kept, under the Act of Parliament, and which the keeper was bound to allow the Commissioners in Lunacy to have access to, was as much a public book as the books of the Stamp Office, or of the India House, which are admitted as evidence as public documents. Sir Frederick also cited cases in support, especially Hyam against Ridgway, a pedigree case, in which it became necessary to establish the time when a particular person was born. A gentleman had attended the mother of the claimant in her confinement, and had made an entry of the fact of the birth in his book; and he being dead, the book was admitted for the purpose of proving the pedigree.

Mr. PETERSDORFF followed on the same side, and quoted the authority of Mr. Roscoe, who laid down the rule that where an entry or declaration is made by a disinterested person, in the course of discharging a professional duty, it is in general evidence after the death of the party making it. Mr. Petersdorff cited the instance that a notice endorsed by a deceased clerk in an attorney's office is evidence of the service of the notice after the death of the party.

Mr. Serjeant WILKINS contended that his objection had not been met. Firstly, as to the book kept by Dr. Millengen being a public book; the only object of it was that it might serve as a check upon himself; and in order to obviate the abuses liable to occur in private lunatic asylums; it was to satisfy the commissioners in case any complaint should be made. The cases referred to by Mr. Petersdorff were not applicable. It was true that an entry of a notice by a deceased clerk was held to be evidence; but evidence of what? of the service of that notice, and not evidence of the contents. Suppose the case put by Sir F. Thesiger, where a medical man had attended a lady during her confinement—had he thought proper to insert in his book, not only that she had been delivered of a child, but that she had been delivered of a bastard, would that have been evidence of the bastardy? So the learned Serjeant did not object to the book being admitted as proof of the fact of this lady having been an inmate in this asylum; it was not evidence of anything more. In the cases quoted, also, the parties were disinterested witnesses; and no witness could have a stronger interest in keeping this lady in his lunatic asylum than Dr. Millengen.

Mr. SOUTHGATE followed on the same side. He argued that it was simply a question of law. He contended, like Mr. Serjeant Wilkins, that all the cases cited on the other side went to show that if in a public document made by a man in the ordinary course of his duties, you find an entry made as to a particular fact done by him, that is evidence of that particular fact, but it is not evidence of any matter in any way collateral to that fact. It did not follow that because a book is directed to be kept by Act of Parliament, that it was therefore evidence—if so, what would be the use of inserting in Acts of Parliament a clause directing that certain books shall be evidence? Was there any such clause in the Lunacy Act? Certainly not. The book kept under that act was a private return made by the keeper of the asylum to the commissioners, who were sworn to secrecy; therefore they who sought to put that book in, sought to do that which the commissioners themselves could not disclose without violating their oaths.

Sir F. THESIGER here said—Dr. Prichard, who was himself one of the commissioners, was examined in 1846 under this very commission.

Mr. SOUTHGATE replied that Dr. Prichard was examined to prove that he saw Mrs. Cumming, and that such and such things took place between him and Mrs. Cumming, but he could not be examined as to any fact which had been told him by Dr. Millengen. Mr. Southgate illustrated the inadmissibility of entries made by deceased parties as evidence of any collateral fact more strikingly and appositely, by stating that, in the pedigree case, the apothecary had entered in his book the fact that he had attended a lady upon the birth of a child—that book was held to be evidence of the fact that he had attended the lady; but, suppose that he had entered that at the time the child was born the lady was insane, would that be evidence of the insanity of that lady? He also observed that in pedigree cases evidence was admitted which was not admitted in other cases—such as entries in family Bibles. Mr. Southgate, lastly, urged that the book was only evidence of the fact that there was a patient in the asylum of the name of Catherine Cumming, and submitted that it was not evidence of anything more, and more particularly as it was impossible to cross-examine as to the reasons for the conclusion to which Dr. Millengen might have come.

The Commissioner did not think the book evidence, as a book, but thought it evidence as the evidence of a medical man who had the charge of Mrs. Cumming at that time. It was open to the observation that you cannot cross-examine him; and also that he was the person in whose legal control she was, and therefore it might be, to a certain extent, a book to justify his own acts.

The Report was then put in, and read from the book kept by Dr. Millengen, and produced by the witness.

There was no entry in it of the visit of Dr. Conolly or Dr. Webster.

Sir F. Thesiger, to the witness.—When the Commissioners of Lunacy attend, do they examine the patients? A. At their discretion.

The Commissioner.—Of course they cannot go minutely into every case—you must not take that entry as evidence of their having gone minutely into the case, but *prima facie* evidence that they see no reason for their discharge.

Sir F. Thesiger then put in the report of the commissioners to the chancellor, produced by the secretary, Mr. Lutwidge, dated July 1st, 1846.

Mr. Serjeant Wilkins inquired whether Dr. Millengen was not a writer of romances. The witness could not tell.

*Sir Alexander Morison* sworn, and examined by Sir F. Thesiger.—Q. You are a physician. A. I am.—Q. And I believe you have been so for a great number of years? A. Yes, a great many years. — Q. And you are a lecturer upon diseases of the mind? A. Yes I am, and have been for a great number of years. — Q. I believe you are also physician to Bethlem Hospital. A. Yes. — Q. Are you physician also to the Surrey County Lunatic Asylum. A. Yes. — Q. And I believe you were also the consulting physician to the Middlesex County Asylum? A. From 1831 to 1848. — Q. And you are author, I believe, of several treatises on mental diseases? A. Yes. — Q. Have you made the subject of diseases of the mind your study for a great number of years? A. I have tried to do so, at least. — Q. In the year 1846, did you, at the desire of Mrs. Cumming's family, visit her in the York House Asylum? A. I did. — Q. Do you recollect the day on which you went? A. To the best of my recollection I went on the 18th and on the 19th of May, and about the 19th or 20th of August. — Q. Upon the first occasion of your seeing Mrs. Cumming, how long do you think you were with her altogether? A. It was not very long the first occasion: she was engaged with two gentlemen—a Mr. Farrer, a solicitor, I think, and his brother. The next visit, which was the next day, I was with her, and should think about an hour.

Mr. Serjeant Wilkins.—Did you say the gentleman's name was Farrer? A. I was told so.

Sir F. Thesiger —I suppose before you saw Mrs. Cumming you had endeavoured to make yourself acquainted with all you could learn of her state of mind? A. Yes I had. Mr. Ince then called upon me, and stated—— Q. He had called and communicated to you particulars which it was necessary for you to know? A. Yes, and previous to going to York House, I had gone, by his desire, with Mr. Johnson, a surgeon, on the 11th of May, to Mrs. Cumming's house, in Belgrave-place, and after knocking and ringing several times, he was admitted, but came out and said the lady would not see anybody. — Q. Was that prior to her being removed to York House? A. Yes. — Q. So that you were unable to see her before she was removed to the asylum? A. I was unable to see her. — Q. Did you also have conversation with Mr. Johnson as to this lady—did he communicate with you? A. Yes. —Q. I suppose your experience would tell you it was necessary you should be possessed of this previous information? A. Of course, to know upon what points to examine the patient. — Q.

On the 19th of May you saw her, and had an interview with her for an hour? A. I should think so—a long interview. — Q. Were you there, in your judgment, sufficiently long to be able to ascertain the state of her mind? A. I was. — Q. What was your opinion of the state of her mind at that time? A. That she was not of sound mind; that she laboured under what I conceived delusions, both with regard to her children and property. — Q. What, in the course of that interview, did she state about her children which you characterize as delusions? A. She stated that they had robbed her, and that her son-in-law had destroyed one or two of her grandchildren, that after death he had varnished over the face of one of them. — Q. Will you allow me to ask which of her sons-in-law had destroyed the children? A. Mr. Ince, I think; that he had destroyed one of Mr. Hooper's, one of the grandchildren. — Q. That her solicitor, you were going on to say. A. That her solicitor had also robbed her, I think she said as to 300*l.* I am not certain as to the sum, but I think it was that. — Q. Did she state anything to you about her husband? A. Yes, she stated that he was of loose habits, and the nurses went to bed with him several times. — Q. Did you know of his being at that time a gentleman very far advanced in life? A. I have seen him; I went to the house to see him, and saw him on that occasion. — Q. Did you go after this interview? A. I went before I went to York House. — Q. What did she say upon the subject of her property? A. That she had been robbed of her property. I asked her what her property consisted of, and she seemed to have some tolerable notion of that; she said she had about some 500*l.* a year, that it would be 1500*l.* if the railroad went through it. — Q. In what state was she as to her dress when you saw her? A. When I saw her at York House she seemed very well dressed, and walked about; no appearance of anything like palsy; she exhibited a great deal of shrewdness. — Q. Do you find, from your experience, that persons who are labouring under what are called monomania, or delusions, are cunning enough to conceal them? A. Oh yes, to conceal them completely, sometimes. — Q. Can persons in that state at times be tutored to conceal their delusions? A. I think so. I have occasionally advised my patients in Bedlam Hospital that such and such delusions would prevent their being liberated, and that they ought to try to get rid of them. They have occasionally tried to get rid of them, and after a while they have returned. We had a remarkable instance there lately of a poor woman who destroyed herself. After a time she got rid of her delusion, so that I put her down in the convalescent list, and soon after she committed suicide. — Q. They are capable of repressing the exhibition of delusions for some time, but they will return? A. Particularly if they are spoken to, and told it injures them their talking of them. — Q. From the interview you had with Mrs. Cumming on that occasion, what was your opinion as to her state of mind? A. I mentioned that I considered her to be of unsound mind. — Q. And I believe you signed a certificate to that effect? A. Perhaps I did; I do not recollect. I did not sign the certificate for her being conveyed to the lunatic asylum. Perhaps I may have been asked my opinion at that time. — Q. Did you attend the inquiry at the Horns Tavern? A. The whole of it; and a wearisome job it was, something like the present. — Q. Was Dr. Pritchard there. A. He was. — Q. Was he examined as a witness? A. He was. — Q. You heard him examined? A. I heard him examined. — Q. Were you examined as a witness yourself? A. I was. — Q. Was Mr. Millengen examined? A. He was. — Q. Was it while the commission was pending or before the commission that you saw Mrs. Cumming again, in the month of August? A. Before the commission. — Q. When you saw her in the month of August, did you converse with her so as to endeavour to ascertain the state of her mind? A. I did. — Q. Were you alone with her on that occasion? A. I could not burden my mind, but I think I saw her alone for a certain part of the time. — Q. Upon that occasion did the same delusions, as you have called them, prevail as upon the former occasions? A. They did; the same ideas prevailed. — Q. And upon that, upon your interview with her, you gave your evidence before the commission upon the former occasion? A. I did, and on hearing the evidence that was brought forward before my examination, a great deal of which was given before the examination of the medical men——

Sir F. Thesiger.—We are both agreed as to this; it seems to have been laid down on high authority that it is not competent to ask the medical gentlemen—having heard the whole of the evidence, what is your opinion as to the state of mind of this party?—because that is a question for the jury, which will account for my abstaining.

Mr. Serjeant Wilkins.—It is the province of the jury.

Sir F. Thesiger.—That is my impression, and that is why I abstain.

The COMMISSIONER.—You have a right to ask a professional man his opinion on a particular case. You can state a case to a professional man, but you have no right to ask him generally his opinion on what he has heard, that is putting him in the position of the jury. If you pick out particular facts in a case, you may ask his opinion on those facts; and that was laid down by the lord chief justice.

A JURYMAN.—But the party may ask a medical man his opinion derived from his own knowledge?

Sir F. THESIGER.—Certainly, I will just put this by way of illustration of the course which is open to us.

The COMMISSIONER.—It used to be always done.

Sir F. THESIGER (to the witness).—Suppose a person has placed his affairs in the hands of an attorney who has conducted himself with the strictest propriety and integrity, and who has from time to time received money, and rendered due and proper account of the money, which has been sanctioned by the person by whom the attorney was employed, and that person should afterwards represent, on several occasions, that the attorney had robbed her or him, I will put it, had left him penniless—had rendered no account whatever, and was rolling in luxury upon the property he had so acquired—would you consider that delusion or not? A. I would consider it delusion, certainly. — Q. In the month of October we hear there was an order from the Lord Chancellor to yourself and Dr. Monro to go down to Brighton to examine this lady? A. There was. — Q. And to take any person with you, and pursue any course of examination you thought proper? — Q. Yes, to go together or separately, as we thought proper. We were to have gone together, but Dr. Monro was seized with erysipelas, and was laid up. — Q. When you went down to Brighton, did you desire to have the assistance of another medical gentleman? A. Yes. — Q. And I believe Dr. King was determined upon? A. Yes, Dr. King. — Q. From what you had heard, did you think it necessary to have some of the police with you when you went to the house? A. Mr. Turner met me there, of course. I went there to see the patient; I had nothing further to do but to be introduced to the patient. In what way I left to others, I could not direct that. Q. Having been admitted to the room, did you address Mrs. Cumming. A. Yes. — Q. Did you tell her for what purpose you had come? A. Yes, I told her the Lord Chancellor understood that she had been unhappy, or some expression similar to that, and that he had wished us to come down and inquire into the state of matters, and I think she said she was not insane. — Q. Mr. Turner was in the room—you wished to have some one there? A. Yes. — Did Mrs. Cumming, when you went into the room, appear to you to be alarmed? A. No, I cannot say so much; she seemed very much as you saw her the other day. — Q. Quiet, was she? A. Yes, she did not rise out of her chair, or make any particular noise, except when her daughter, Mrs. Ince, who was there, wished to come in, and she said "no, never," in an angry tone. — Q. Will you be good enough to state what questions you put for the purpose of ascertaining the state of her mind? A. There were some questions I was led to put which I thought would throw light upon the state of her mind. In particular I asked her, "When you were in Herbert Villa, what was it made you call out at midnight for the police?" Her answer was, "It was because my daughter was strangling me." That was one question. — Q. Did you ask anything else? A. Yes, I asked her something else. I asked her if she had seen her daughter, or if she did not wish to see her daughter? and she told me she had not or would not. — Q. Did she say why? A. I do not think at that time. Then I asked her why she had left her house in Queen's-place, or terrace? I think she said, "Because they wished to poison me. They put poison in my tea." — Q. Did she say who it was who wished to poison her? A. No, she did not. — Q. Did you ask her why she had moved from place to place? A. Perhaps; I do not recollect. There was another question I asked her, by-the-bye. I asked her, first, who had advised her to depart from the compromise of 1846? She said it was her lawyer's advice. I then asked her if she had made a will? and she said she had. I asked her if she had left anything to her daughters? She said no; she was very angry, and expressed great anger against them. Well, since your grand-daughters have not offended you, have you left anything to them? No, I have left it to those who deserve it, or who have befriended me. I asked her who they were? She would not name them. I asked her several questions as to her property, but she either would not answer or could not. Had she sold any of her property? What did her property consist of? and several questions of that kind. — Q. You put, for instance, the question to her, "Have you sold any of your property?" A. Yes. — Q. What did she do or say upon that? A. She did not give any answer. —

Q. She was silent? A. Yes. — Q. Have you asked her what her property consisted of? A. Yes, I asked her what her property consisted of, who her tenants were, and several questions of that description; to all of which she gave me no answer. She asked me, on one occasion, "Am I obliged to answer that question?" I said, No, you are not obliged to answer any question, but such questions as you do not answer, I shall be obliged to state to the Lord Chancellor. — Q. Was she during the time excited? A. I do not know exactly what to call excited; if any, she was uncomfortable, and did not like to be questioned in that way, but she did not show any violent excitement, not such as I am accustomed to sometimes. — Q. Was it your opinion upon that occasion that she was a person of sound or unsound mind? A. I was of opinion she was of unsound mind. — Q. Did it appear to you that the same delusions which she had displayed in the year 1846 continued at that time? A. With some variation. That unnatural antipathy towards her children and grandchildren continued, and that disposition to delusions continued. For instance, supposing her daughter, who was miles off, strangling her at the time she called for the police. These appeared to me decisive marks of unsound mind. — Q. Is that a common form of insanity, persons dreading that they are to be murdered? A. Almost in all recent cases that is one of the indications. — Q. Is the destruction of the natural affections a common form of insanity? A. A very common form; it is one of the most common forms that people's affections have changed; and one of the most decided signs of returning sanity is a return of the natural affections. — Q. Do you find, when insanity prevails in persons advanced in life, they are more or less likely to recover than in the case of younger patients. A. Much less likely. — Q. From what you have seen and know of Mrs. Cumming, you think she is a person who could be tutored to conceal her delusion for a certain period? A. From the shrewdness which she had sometimes exhibited, I should think something might be made of her in that way, if you visited her a dozen times, day after day, repeating a story in the way you wish to have it done. — Q. You think it would impress it on her? A. I think she is a subject for that, or to alter the character of the delusion. For instance, I think she might be made to alter the delusion of being strangled by her daughter, or she might be made to say that was a dream, or something of that kind. I think she is a person who might be worked upon in that way. — Q. Is it your present opinion that she is a person of unsound mind, and incapable of taking care of herself and her property? A. It is decidedly.

Cross-examined by Mr. Serjeant Wilkins.—Q. Is your recollection very distinct of what took place at Brighton? — A. Not very distinct, but quite distinct as to that delusion, perfectly distinct. — Q. You say you asked her why it was that she called for the aid of the police on that night? A. Yes. — Q. And you said she told you one of her daughters was attempting to strangle her? A. Yes. — Q. May it by possibility be that you have made a mistake, and that what she said was, that one of her servants was trying to strangle her? A. Certainly not; she said one of her daughters—her daughter. — Q. Your first interview was in May, 1846, I think you say? A. Yes. — Q. Had you, before seeing her in 1846, received a statement from Mr. Ince? A. I had. — Q. A somewhat lengthened statement, I think? A. I cannot say that at all. I do not think it was a lengthened statement. He had called upon me, and told me certain circumstances respecting this lady. — Q. And told you some of the delusions under which she laboured? A. Yes; told me she had accused him of robbing her, and those other circumstances. — Q. Did he tell you that she had accused him of robbing her of a silver bread-basket? A. I do not recollect that. — Q. Did he give you a history of the transaction respecting the silver bread-basket and some silver salt-cellars? A. I do not recollect. I heard it at the trial. — Q. You also had an interview with Captain Cumming before you went, had you not? A. Yes. — Q. Had you a conversation with Mr. Johnson before you went to the Asylum? A. No. I do not think it, before then; when I went with him, on the 11th of May, to the house. I do not think I saw Mr. Johnson after that. — Q. You say that she told you her income was 500*l.* or 600*l.* a year, but that it would be increased to 1500*l.* if the railway was carried through her property? A. Yes. — Q. Did she not state that the fact of the railway passing through her property would so enhance its value as to raise it to that amount? A. No, I do not think she did, but it came to that in round terms. — Q. My learned friend, Sir F. Thesiger, asked you just now, supposing her attorney to have acted with strict propriety, and that the client should afterwards think that that attorney had robbed her, (which was in effect what my learned friend said,) would you call that a delusion, and your answer was, you would? A. Yes. —

Q. Supposing her naturally of a suspicious disposition, and stingy in her money transactions, and suppose that attorney during the whole of his transactions with her estate had only rendered her one written account, and suppose that attorney to be on intimate terms with her son-in-law, who she suspected to have robbed her, would you have treated her impression as a mistake or as a delusion? A. That is a hypothetical case. — Q. I put it to you as a hypothetical case. My learned friend Sir F. Thesiger put a hypothetical case to you, and that is my hypothesis. A. I would require to make particular inquiries myself, not exactly as you state it. I should wish to inquire whether it really was true that this attorney had done any wrong to her. — Q. You are to assume that—you are to assume that the attorney (I do not say that he had done any wrong) but you are to assume that that attorney had the management of her affairs for a considerable length of time; that he received her rents; that he had advanced her monies; that all her money transactions passed through his hands; and that he never gave her more than one written statement of the accounts between them, and that before the accounts concluded; supposing all that? A. It depends upon the length of time the account was standing, for it might not be a very long time. — Q. My hypothesis is quite as tenable, and quite as answerable, as that of my friend.

Sir F. Thesiger. It is a very long one.

The Witness. It might be a mistake in the woman, but as it was stated to me, it appeared to be delusion.

Mr. Serjeant Wilkins. Have you not found among sane persons a great many persons who are of a very suspicious character? A. I do not know that I have found many among sane persons. I have not had intercourse with many persons of suspicious temper. I do not know many persons of a suspicious temper; very few indeed. — Q. You are a very happy man: you do not know any? A. No; I cannot say I am acquainted with any of what I would call a suspicious temper. — Q. Would a proneness to suspicion be an index or a symptom of insanity? A. Not without other symptoms. — Q. Did you make any inquiries at all as to the moral character of Captain Cumming? A. No, none at all. — Q. Did you make any inquiries as to whether upon the night in question, who had attempted to throttle or strangle her when she called for the police? A. No; certainly not. — Q. Did you make inquiries as to what were the grounds on which she suspected her son-in-law of robbing her? A. Mrs. Cumming herself? — Q. Yes, or of any other person. Did you take any pains to ascertain whether there were any grounds for this charge or not? A. No. — Q. In fact, you assumed it to be a delusion? A. I assumed it from the manner of the patient, and other circumstances. — Q. Would you call this species of insanity monomania? A. Yes; I would; monomania founded on unnatural hatred of her children, leading to a great variety of delusions. — Q. Would you think that hatred of offspring, *per se*, is a proof of insanity? A. If it extends to children and grand-children, I should think it a strong symptom of it. I will tell you; there is a case lately decided in Scotland; but perhaps you will not go so far north? — Q No; I would rather have your opinion in England. A. But I may mention that, in that case, the hatred of the parent was considered sufficient to do away with the will. — Q. That is the case of Dew and Clarke, is it not? A. No; that is the case of Fraser. — Q. That case has nothing to do with this, has it? A. No; but I mention it to show you that unnatural hatred alone has been held to be sufficient. — Q. Never mind about that. My question to you is this, do you think that the hatred of offspring, and of the offspring of offspring, is proof positive of insanity? A. I think it is. — Q. Do you think that ingratitude and a perseverance in cruelty and neglect would be unnaturally productive of hatred? A. I think it would naturally produce hatred. — Q. In the mind of a sane person? A. In the mind of a sane person, yes. — Q. Had you ascertained that Mrs. Hooper had married very much beneath her, and against her mother's consent? A. I have heard so. — Q. Had you heard that at the time? A. I do not recollect. A great many things appeared upon the trial that I could not now separate from what I had heard before. — Q. Had you heard of the marriage which she opposed? A. I had heard that she opposed the marriage of one of her children, Mrs. Hooper. — Q. Did you hear also that Mrs. Ince aided and assisted in that marriage. A. I heard Mrs. Ince state it yesterday here; I did not hear it before. — Q. When Mrs. Cumming expressed her dislike to Mrs. Ince, did she put that case? Did she not refer to her alliance with her sister, in disobedience of, and frustrating, her wishes? A. She might.

Sir F. Thesiger.—I think you do not understand the question.

Mr. Serjeant Wilkins.—The question was, whether Mrs. Cumming referred to Mrs. Ince having assisted Mrs. Hooper in the marriage, as a ground of her objection?

By the Commissioner.—Did Mrs. Ince give any reason for her aversion to Mrs. Hooper? A. She does give that ground, but I do not remember when she did give it.

Mr. Serjeant Wilkins.—Did she not complain of their repeated acts of cruelty in seeking to confine her, and treat her as a lunatic? A. She has since, but not prior to that hatred. — Q. Did she not then? A. No. — Q. Prior to what? A. Prior to her being sent to York House. — Q. Prior to your being sent to Brighton? A. No; she never complained to me. —Q. You could hardly call a decay of intellect insanity, would you? A. We call imbecility insanity, unsound mind. — Q. Would you call a decay of intellect from age insanity? A. We call imbecility, and unable to manage her affairs. — Q. In old persons? A. If unable to manage affairs. — Q. Do you call the ordinary decay of mental faculties, the necessary result of age, such imbecility as to render a person unfit to manage his affairs? A. Certainly not; not until it amounts to senile imbecility. — Q. That is second childishness and mere oblivion. Did she complain to you of the cruelty of her children in their constant efforts to confine her? A. No. — Q. On no occasion? A. Not to my knowledge—not to my recollection. More of robbing her and wishing to destroy her, not of confining her. — Q. Did you take any pains at all to inquire into the case of poisoning? A. Not further than hearing what she said. — Q. Did she not tell you Dr. Barnes had analyzed the poison? A. No; she did not. I have heard that since, but she did not tell me. — Q. Did she tell you that poison was given in a cup, but being analyzed by Dr. Barnes, four years ago, was ascertained to contain oxalic acid? A. No; she never said a word about it. — Q. There is your own report, "My Lord, having visited Mrs. Cumming, for the purpose of ascertaining the state of her mind, Edward Thomas Munro on the 6th or 8th instant, and Sir Alexander Morison on the 27th, we are of opinion she is of unsound mind, on the following grounds, namely, that she believed her daughters to be her enemies, and plotting against her; that she has every reason to believe that one of them attempted to strangle her, and that poison was given her in a cup, which had been analyzed by Dr. Barnes, five years ago, and was ascertained to contain oxalic acid, and of which a fowl was afterwards killed, &c.?" A. That about the poison is Dr. Monro's altogether. She said nothing to me about poison. — Q. Did you see that report? A. Yes.

Sir F. Thesiger.—You are not regular in introducing it in this way, for it states that Dr. Monro visited her on the 8th, and Sir Alexander Morison on the 27th, and this is the joint report of these two gentlemen.

Mr. Serjeant Wilkins.—The interruption is as unfair as it is irregular, and does not explain away the difficulty, and I again ask Sir Alexander Morison if he put his signature to that certificate. A. I did, and Dr. Monro will explain it to you. I have explained it to you. My explanation is, that she stated to me that poison was attempted to be given to her. It was explained to Dr. Monro, and that is in our joint report. — Q. Is this the instance of poisoning which she explained to you? A. I cannot tell; she said she had been attempted to be poisoned. — Q. Is this the instance explained to you? A. I cannot tell; I suppose it is. — Q. Did not you ask for any explanation as to this circumstance of the poisoning, and under what circumstances? A. No; I did not.

The Commissioner.—Were you present when Dr. Monro was present at the examination? A. No.

Mr. Serjeant Wilkins.—Did she say to Mr. Turner in your presence that the poison was put in a tea-cup, and that she could prove it by medical men? A. I do not recollect proving it by medical men. I recollect she said she was attempted to be poisoned in her tea, but I have no recollection of proving it by medical men.— Q. Did you make any inquiry at all as to the nature of the poison? A. No.

*Dr. William King*, sworn, and examined by Mr. Petersdorff.—Q. I believe you are a practising physician at Brighton? A. Yes. — Q. Have you practised there a great number of years? A. Yes. — Q. Are you now, or were you at one time, the proprietor of Ringmer Lunatic Asylum? A. Yes. — Q. You have had, I believe, a great deal of experience in cases of lunacy and unsound mind? A. Yes, for a man who does not profess that line of practice, I have had a good deal of experience. — Q. Do you remember, in the month of October, an application being made to you as to seeing Mrs. Cumming? A. On Monday, the 27th of October. — Q. Was any explanation given to you respecting the order from the lord chancellor for that purpose? A. Yes; Mr. Turner called upon me and explained why he wanted me, and by what authority he was acting. — Q. Did you see Sir Alexander Morison at that time? A. Yes. — Q. Had you any conversation with Sir Alexander Morison before you saw Mrs. Cumming, as to her state of mind? A. Not particularly. — Q. Did you go to Mrs. Cumming? A. Yes. — Q. Was the door ultimately forced open by the police? A. Yes, by Mr. Chase. — Q. Did you go into the room as soon as the door was forced open? A. No; Mr. Turner and Sir Alexander Morison went in first of all. — Q. Did you go into the room or not during the time they were there? A. Not while they were in the room, not till Sir Alexander Morison came out. — Q. And when Sir Alexander came out you went in? A. Yes; there was great opposition made. — Q. Great opposition for anybody to go in? A. Yes; for Mr. Chase was nearly thrown down stairs, and might have been killed, and a more infamous piece of business never was. — Q. Did you see a Mr. Jones there, or James, at that time? A. Mr. James I did; it was Mr. James who grappled with the chief officer, who was nearly being thrown down and breaking his neck, and that is the consequence of having orders of the lord chancellor, and if that is not a crime, I do not know what is. — Q. You say, at last, you got in? A. Yes, when Sir Alexander came out I went in. — Q. When you went into the room, there was Mr. Turner, Mrs Cumming, and anybody else? A. Mrs. Watson—the four. — Q. Now state what passed between them and Mrs. Cumming in your presence? (The witness refers to a paper.) A. I began, first of all, asking her about those delightful cats that have been mentioned. — Q. What did she say about the cats? A. She said she was fond of cats, and I asked her if she kept them in her room? she said she never kept them in her room—always in the kitchen; and I said, was your own clean and comfortable? she said, always very clean and very comfortable. Then she said she took them in a carriage with her, and made use of a general expression—two or three dozen at a time, and that they were fed in her room—not fed in the room—not kept in the room—and the room was always clean, and the cats were always kept in the kitchen; then I asked her about her daughters, and she said she had a great hatred to them because they had attempted to poison her, or strangle her, and that Mrs. Ince carried her in her arms, and attempted to strangle her. She did not say definitely which it was that attempted to poison her, but that they had attempted to poison her; and I asked what proof she had of that: she said they put poison into her tea—that this was given to the cats—that the cats would not touch it, and that it was given to a fowl, and that the fowl died of it: she said her daughters had treated her disrespectfully, and that they had married below their rank; she did not say that the one had married below rank, but they had, and that was her reason of hatred to them; and then I endeavoured to pacify, and to make her mild upon that point, and asked her to see her daughter, and whether she would provide for her in her will after what had happened; she said at first she would, and after that she would not. There was a certain incoherence and indefiniteness about her: she also said that it was her daughters that induced their father to send her to an asylum during his lifetime: she laid the blame upon them. Then I asked her about her husband, what sort of a person he was, and whether he was kind, and so on, to her; she said that he was a gentleman, and a man of character, and that he was very kind to her, and treated her well; but, after some little time, she began to abuse, and to accuse him of what has been mentioned in evidence so often, accusing him of having had intercourse with her servants, and that on one occasion she had caught him in the fact. Then I asked her something about her grandchildren, and she said she had some grandchildren, and that two of them had died; but she professed ignorance as to the nature of their diseases, or the cause of their death. I then asked her how she came to Brighton—whether she came by rail, or in what way she came; she said, at first, she came direct from London in a post-chaise, and not by rail; but afterwards, in the course of conversation, she said she had been at Worthing before she came to Brighton, and she said she had seen Dr. Barnes at Worthing—Dr. Barnes from London—she did not enter into particulars then. I asked her about Mr. Thorn, the solicitor, how it was she dismissed him; and she said it was because he did not attend to her. I asked her if she was aware that he called upon her, and had been refused admittance; she said not. I asked her how long her servant, Mrs. Watson, had lived with her, knowing that she had seven weeks; then I asked her a few questions about her property, whether she knew what property she had, and what had been done with it; and she said no, she did not know what her property was—she did not know what had been sold—when it had been sold: on asking her about signing papers and so on, she said she had signed papers, but she did not know their contents—that is the substance of the conversation I had with her on different points. Then, in judging of the state of mind of persons, you take into consideration the whole manner, and tone, and expression, and particularly the expression of the eye, and the mode in which they make their answers, and their general gestures and behaviour; it is not simply what they say, but how they say it. — Q. What was the conclusion at which you arrived from your observation? A. My conclusion was that her mind was not sound, and that knowing nothing at all about her property, what she had sold or not, or what the contents of the papers were that she had signed, that she was not competent to manage her property. — Q. Was your attention directed, or did you make inquiries with respect to her bodily health at that time? A. I was not requested to do that, but I called on the Wednesday for that purpose. I understood she was infirm; she was sitting in a chair at the bed, with her face towards the bed, her back to the wall, and the fireplace on her left-hand side. As to those particular points about the cats and the papers, and her daughters, a good deal would depend upon the facts, whether they were true or not, as to whether she was telling lies, or whether she was under a delusion. — Q. You said you went on the following day—I asked if you had ascertained the state of her bodily health? A. No; this was on the Monday. I was going to state that I went on the Wednesday; this was on the Monday; I considered that my office was over. — Q. Did you, on the Wednesday, ascertain the state of her health? A. I went into the house and walked up stairs.

Mr. Petersdorff.—Now, on the Wednesday, what took place? A. On the Wednesday I called in the afternoon, and then I found a person who would not give me his name, and who afterwards proved to be Mr. Ellis, and I requested to see her. The door was locked, and they would not admit me, and I said I came from the lord chancellor, or by the authority of the lord chancellor, which I considered to be the case, and I then said, "Well, I have done my duty. I have done what I think right, and you have done what you think right, and as I cannot see her I shall walk out of the house. — Q. You did not see her on the Wednesday, then? A. No. — Q. Did you, at any time afterwards, see her to ascertain the state of her health? A. No, not to ascertain the state of her health. I saw her on the Thursday, but I am sorry now, from what I have heard since I came up here, I did not see her and ascertain the state of her health; I was not aware that she was paralysed, or semi-paralysed, in the legs, otherwise on the next day, Thursday, on which she was carried away, I should have wished to be aware of that circumstance: but it has been stated she went out in her carriage every day, therefore if she was put into a common carriage, and it might be very improper to carry her from London in that state, without any proper motive for it; but to carry her to London from Brighton, under the circumstances, was quite another question. Then, on the Thursday, I went and called on Mr. Turner, at his lodgings, and told him what had passed. — Q. That was after you had been refused? A. Yes, on the Wednesday, after I had been refused entrance, I went and informed him of the circumstance, and we went together to Mr. Verral, the clerk to the magistrates.

Sir F. Thesiger.—You need not go into that case.

The Commissioner.—You were refused on that occasion? A. Yes; then, on the Thursday morning, I went to Mr. Chase. I had a note from Mr. Turner, to say that he was going to London, and I understood that they would not allow the nurse to take her away who was sent down from London. I therefore went to Mr. Chase, the chief officer of police, and I said this is a very peculiar case; here the lord chancellor has sent down to examine this woman—she is pronounced to be insane, she ought therefore to be removed to an asylum—the nurse has authority to remove her, and we may consider that authority as coming from the lord chancellor. There are people in the house who will not allow her to go; and I said, will you go up to the house, and stand by, and keep the peace while the woman performs her duty, for Mr. Verral, the clerk to the magistrates, had told us she was authorized to do so; he took the act down to consult it—perhaps I was a little out of my line there, but I did think it was abominable. — Q. What did you do? A. Then Mr. Chase and I went up together, and went into the house, and called all the people in the house together, and there we found the nurse from the asylum, with her brother, older than she was. The nurse was a very sensible, quiet, judicious woman—we called them together, and Mr. Ellis and Mr. James into a lower parlour, and then I said this woman has got authority to take poor Mrs. Cumming away from this house; Mr. Chase, an officer of the police, is come to protect her in so doing, and she will give her orders as to what is to be done, and how it is to be done. Well, then we went up stairs, and found the door again fastened inside, and they spoke; this woman spoke to the persons inside to desire them to open the door, and they refused to do it—at least they did not say anything—the door was not opened, and, after waiting a certain time, she then desired Mr. Chase to open the door. Mr. Chase put his back against the door, and opened the door, and then it appeared to him they had put on a very strong bolt, and in breaking open the door the architrave was torn away, from the force used; there we found Mrs. Watson and another woman, who would not give her name; but it appears since it was Mrs. Hutchinson; then the nurse said she was come to take her away, and she should do so. They did not offer any physical resistance, but said she was not fit to go—that she wanted to be dressed, and so on; and I said, of course they will dress her properly for the journey; and I made some observations as to Mrs. Hutchinson being in the room. She said she hoped they would not turn her out of the room, because she was a friend and an acquaintance. I did not know who she was, and I thought, out of feeling of delicacy to this poor woman, there was no occasion, provided she did not obstruct; and then I went out of the room, and looked in occasionally, to satisfy myself whether she was obstructing or not; however, they were a long time dressing her, partly from her infirmity of body, and so on; and then at last it was done, and Mr. Chase said, there will be no more obstruction; I shall go, and I will send an officer down to the station to go to London with them, and I will telegraph to London that she is coming by this train, at a quarter to two; it was the first train, and that was done, and a cab was got, and they went down. She was dressed, and her brother and Mrs. Cumming and the policeman were put inside the fly, and one policeman went outside the fly, and they set off—there was no room for me, so I thought it my duty to see them safe out of town, and I went down in another fly; and when I got to the station, I found them upon the platform and the train was just going to start; and the nurse said her brother was going to start, and that he had gone for the tickets. She had not got the tickets—the train would start without them—and wanted to know what was to become of them. — Q. At this time, did Mrs. Cumming appear to be in sufficient bodily health to perform the journey without injury? A. I should say so, certainly, but I would make the distinction I did before; I had been consulted as to the necessity of sending her unnecessarily out of London, that would have been one thing, but the sending her back to London would be another; and then I found the police officer who went up with them.

Cross-examined by Mr. James. — Q. In whose presence did you examine her? A. In the presence of Mrs. Watson and Mr. Turner.—Q. Did Mr. Turner examine her? or perhaps a more proper expression would be, did he cross-examine her in your presence? A. No; he asked her some questions about her property. — Q. This long statement that we have had of questions and answers, did they transpire in your presence? A. Not the whole of it, because part of it occurred in Sir A. Morison's presence. — Q. While you were examining her, did Mr. Turner put questions? A. He put questions at the latter end of my examination. — Q. So that I mean she was undergoing examination by you and by Mr. Turner at the same time in the course of the same interview? A. In the same interview Mr. Turner asked some questions about her property which I could not have asked her about. — Q. Were you aware that she had stated to Sir Alexander Morison that her property, her rental, was four or five hundred, or five or six hundred a-year, and that she expected her property would be wanted in the event of the railway passing through it, and that it would be 1500*l.* a-year if the railway went through it? A. I was not aware of that. — Q. Had you any conversation with Sir Alexander Morison before you went in, and subjected her to examination? A. No.—Q. Mr. Turner stated that she felt an indignation at you when you went in? A. No indignation, but about my saying that her fondness for cats was rather strange. — Q. Is it strange for a person to be fond of cats? A. I think so—to that extent it is a matter of degree. — Q. Is it strange to take a cat for a drive? A. Not a cat. — Q. Did you ever know ladies taking poodle dogs out with them? A. Yes, but not cats. — Q. Then you think the distinction is between taking a dog for a drive and taking a cat? A. I think there is a great distinction. — Q. That which might be evidence of soundness in taking a dog for a drive would be evidence of unsoundness in taking a cat for a drive? A. You know that a cat will not follow. — Q. Perhaps that is the reason for taking her in the carriage: you would make that distinction? A. I think taking a dog is a very great distinction. — Q. You have given your opinion that there is a distinction as to sanity between taking a cat for a ride and taking a dog for a ride? — A. There is a distinction; we will not say what. — Q. There is nothing strange in taking a dog for a drive? A. No. — Q. What should you say of taking two dogs? A. That is a matter of arithmetic. — Q. I ask you, what should you say of a lady taking two dogs? A. I say that it is a matter of arithmetic. I should not say anything; that is no business of mine. — Q. Supposing you were called in to examine a lady upon a commission as to her sanity, it being stated that she had taken out two dogs for a drive, what should you say? A. That she took two dogs. — Q. Would you say it was strange? A. No, I should not. — Q. What do you say as to three? A. It would be approximating. — Q. Do you think that would be strange? A. That would be very unusual, three dogs. — Q. What would you say as to four? A. That would be still more unusual. — Q. Would it be strange? A. I think four would: a thing is strange which is unusual. — Q. You think a person may not have so much affection for a cat as for a dog in a sound state of mind? A. To be sure they may. — Q. You think they may. How many lap-dogs have you known kept by ladies in a drawing-room? A. I never knew of more than one lap-dog kept by a lady. — Q. In speaking of cats, do you not know that at Brighton she had not the cats in the bed-room, and that they had been kept in the kitchen? A. I know nothing about it.—Q. Did you find any cats in the bed-room? A. No.—Come, Dr. King, I ask you to give me a candid answer. Have you not since ascertained that at that time what she stated was quite correct, that she kept the cats in the kitchen? A. No; I know nothing about it.—Q. Did you see any cats at all? A. No.—Q. You had not ascertained that she did not in this case keep them in the parlour, but that she had kept the cats in the kitchen? A. No, I do not know that; but her assertion to me was, that she never had kept them in her bed-room. — Q. I ask you, did you not point her attention to the circumstance? A. Not a bit.—Q. Did you not put the question—Do you keep cats in your bed-room? A. No, I did not. — Q. What did you say? A. I asked her if she was very fond of cats, and whether she had not kept cats, and whether she had kept them in her bed-room; it was in the preter-pluperfect tense. — Q. You did not apply the question to the present tense at Brighton? A. No. — Q. That you then put down as strange? A. Yes. — Q. Do you think that if a lady at her age, without society, and perhaps with some suspicion about her servants—do you think it a test of insanity that five cats were kept in her bed-room? A. I think it a very suspicious point, but one point alone will not prove insanity. — Q. Suppose you were called in, and a lady had done nothing more than keep five cats in her room, would you say that she was unsound? A. Not upon that point alone. — Q. I am putting an hypothetical case? A. It is no use going into hypothesis, we are upon matters of fact.—Q. I am asking you, if you were called upon to attend a lady to give an opinion of the soundness of her mind, at her age, without society, kept to herself, having suspicions of her servants, perhaps—I ask you, if you would say it was a test of insanity that she kept five cats? A. She was not a woman without society. — Q. Had you known her from 1846 to 1851? A. No, I had not. — Q. Be kind enough to answer my question. Assuming a lady of her age, without society, and not liking to trust her cats to her servants, would you give it as your opinion that that was a test of insanity that she kept five cats in her bed-room? A. I think it would be a very suspicious circumstance. — Q. Would you say it was a test of insanity? A. No, not by itself.— Q. You have spoken of her children? A. Yes. — Q. And her aversion to her children. I think you said she spoke in disrespectful terms of her children, and treated them disrespectfully? A. Yes. — Q. Have you known instances of the soundest people, where a son has married beneath his rank, or a daughter, that both the father and the mother had refused to see either? A. I have known an instance of a man marrying beneath his rank, and his father and mother wishing to see their child. — Q. Have you known the other? A. Never. — Q. It has never occurred in your experience? A. It has never occurred to me. — Q. Not to you personally. I am not assuming that you married below your rank, or that any of your sons or daughters have done so. A. That is what I mean, that generally I am not acquainted with such a fact. — Q. Would you say that dislike to a child, or a desire never to see her, or even to leave property to her, was a test of insanity, where a son or a daughter had married below their rank? A. Not a test independently.—Q. What would you say of that parent, 73 years of age, who told you that she would never see her child, because she married below her position in society, and would not leave her a farthing, would you say that person is insane? A. I would say that they were unsound to have such a mind and disposition. — Q. I am putting the case of parents who said they never would see a daughter who had married below her condition in life, and would not leave her any property. A. That by itself is by no means a proper sentiment, certainly. — Q. Would it be a test of unsound mind? A. It would not be a test of unsound mind; it might be a crime, and crime is not insanity. — Q. What crime? A. I should think it a great crime, but still not an insane act. — Q. Do not insane persons entertain much more strong feelings than others, and manifest aversion in a different manner—is not that so? A. Yes. — Q. Might not that which would produce aversion in the mind of one person of sound mind not produce it in the mind of another? A. Yes.—Q. Then if a person says, "I will not leave my daughter anything," does not that manifest an aversion, and is it not consistent with a sound mind? A. Yes. — Q. Perfectly sound? A. That might or might not, but it would be no test in itself. — Q. Might it not be perfectly sound? A. Yes, because it would be the act of a villain. — Q. And what? A. An unprincipled man. — Q. But every villain is not insane, do you think? A. No, I say there is that difference. — Q. Might a sound person, of thoroughly sound mind, entertain an aversion to his child, and say he will never see his child, or leave him a farthing, because he has married below his rank? A. Yes; because he might be an unprincipled man, it would not follow that he was insane for that.— Q. Do you think the mere unforgiving spirit to a child having married below her rank is any test of villany? A. It is a test of an improper principle in the mind of a person. — Q. I suppose you have heard of parents' wills, and cutting off their children with a shilling? A. Yes. — Q. From causes more slight than marriage below their position? A. Yes. Q. Now we will take the poison. Have you taken any pains to ascertain whether there was any truth in the statement about the fowl? A. No; I do not see how the stuff is to be got into the mouth of the fowl. — Q. Fowls have mouths, have they not? A. Somebody must have taken it, and poured it down the fowl's throat. — Q. Will fowls pick up sugar of lead with their food? A. Yes; but this was lead solution. — Q. Does oxalic acid very much resemble Epsom salts? A. The crystal is different. The one has been sold for the other. — Q. I suppose oxalic acid thrown down, if a fowl takes it, will kill it? A. Yes, if he had enough of it? — Q. You have taken no trouble to ascertain whether there were any facts upon which that statement of hers was founded? Did you ever see Dr. Barnes upon that? A. No. — Q. She stated to you that Dr. Barnes had analysed it? A. Yes. — Q. Did you ever see Dr. Barnes before you pronounced it a delusion? A. No. — Q. But you pronounced it a delusion at once without any inquiry? A. An improbability of that kind does not require to be inquired after. This court is the proper place to ascertain the facts. They will hear the pros and cons, and the facts and the opinions. — Q. You found that a delusion, without making any inquiry into the existence of the facts? A. Yes, I tried to ascertain the fact. She gave me no evidence to go upon. — Q. Did she not tell you Dr. Barnes had analysed it? A. No. — Q. Do you mean to state that? A. I do not remember it. — Q. Will you say she did not? A. I speak to the best of my memory. I do not remember that Dr. Barnes saw her at Worthing. — Q. Have you a sufficiently accurate recollection to enable you to state that Dr. Barnes had analysed it? A. I do not remember that. — Q. Did she tell you her medical man had analysed it? A. I do not think she did; she merely stated that a fowl had been poisoned by it. — Q. Is Mr. Turner's statement correct—he states that? A. He may have recollected, and I may not. — Q. You say that you were not aware of her physical infirmities at the time you first saw her? A. No. — Q. When did you become aware of them? A. Not till I entered this house. I did not know she had paralysis of the bladder, nor did I know that she had paralysis of the lower extremities. — Q. Did you make an affidavit, in which you stated that, from your examination of the said Catherine Cumming, it appeared to you that she might with propriety and safety be removed to London to an asylum. Did you make that affidavit? A. So she might. — Q. Did you make that upon your first interview? A. I do not know when that affidavit was made. — Q. Just remember. Did you not state that before you were aware of her physical infirmities? A. Yes; there was nothing, in my opinion, that I was aware of, that could prevent her being removed to London. — Q. You became afterwards aware of her physical infirmities? A. Yes. — Q. Did you assist the parties in putting her into the fly? A. No. I was there; I was present, but I did not assist. — Q. Who were putting her into the fly—the policemen? A. The nurse and her brother, and one of the policemen assisted. — Q. Did you push the poor old lady behind, and say, "You men do not know how to put a woman into a fly?" A. I believe I did say something of that kind, because I thought she ought to be lifted in. — Q. Was she screaming from pain at the time?

A. She did complain, now you mention it. — Q. Did she scream? A. I do not know whether I may call it a scream. — Q. You know what a scream is? A. She complained—the nurse ought to have told me she was in that state. — Q. Did you push the old lady behind and say, "You do not know how to put a woman into a fly?" A. I merely protected her. — Q. Did you say that? A. It is very possible I might have said it, because I thought they were very stupid, especially the nurse's brother. — Q. Did you use that expression, "You men do not know how to put a woman into a fly"? A. I dare say I might; I think it very likely I did so. — Q. She expressed a desire, did she not—an entreaty, in fact, not to be sent by the railway? A. No; but Mrs. Hutchinson did; she said that being a lady, she ought to be sent like a lady. — Q. Did she say she had never been by a railway before, but always travelled by the road? A. I am not quite positive; but now you suggest that idea, it is possible she might have said that. — Q. What authority had you under this order? A. I had no authority, and did not give any commands; it was the nurse that did it all. — Q. What did you find she was labouring under, or what did you ascertain to be the extent of her physical infirmity, when you did ascertain it—on the third day? A. I did not ascertain it. — Q. Do you not know now? A. No; only from what I have heard since I came here. If they had gone about the thing properly, and given us proper information, we should have known what to have done. — Q. Did you not swear that it appeared from your examination of her that she might with propriety be removed to the lunatic asylum? A. I saw no objection to it. — Q. Did you examine her? A. I did not examine the state of her bladder, nor the state of her lower extremities; but I was convinced that she might be safely removed; and she was safely removed. — Q. You say, from my examination—did you examine her? A. Yes—so far the word examination has very different meanings. I did not inquire into the state of her lower extremities. If they had allowed me to see her on the Wednesday, this might have come out, and then I should have known. — Q. Did she request you to leave the room while she was dressing? A. I think she did, and so did Mrs. Hutchinson. I do not know whether it was a request, but I left the room immediately. — Q. Did not Mrs. Cumming request you to leave the room while she was dressing? A. I believe she did. — Q. Did she not appeal to your delicacy: did she not beg you to leave the room? A. And I did leave it. — Q. Did she not appeal to you? A. She did. — Q. I believe she appealed to you more than once before you did leave the room. A. No; I do not think that. — Q. Did she not appeal to you more than once? A. I did not leave in consequence of the repetition of it, but I left as soon as I found the persons there were beginning to dress her. — Q. Did you examine her on the Wednesday? A. They would not let me. — Q Did you see the certificate of Dr. Hale that she was not in a fit state to be removed? A. No. — Q. Were you not informed that he had given a certificate that she was not in a fit state to be removed to a lunatic asylum? A. I was informed that somebody had. — Q. I must press the question. Were you not informed that Dr. Hale? A. I thought it was Dr. Barnes. — Q. Were you not informed before you assisted her into that fly, to be taken to the railway, that Dr. Hale had given a certificate that the lady was not in a fit state to be removed? A. No; but when the nurse was going to remove her, Mr. Ellis said, "Now you will recollect you do all this at your peril." — Q. You state you thought it was Dr. Barnes; were you informed of the fact of some medical man having given a certificate? A. I understood that some person had been to see her, and said that she was not in a fit state to be removed. — Q. You said you thought it was Dr. Barnes? A. I had an idea that it was so. — Q. Was not that stated to you before you lifted her into the fly? A. No. — Q. When was it stated? A. I do not know; I think it was Mr. Turner who mentioned it. — Q. When? A. If he mentioned it, it was on the Wednesday afternoon. — Q. Now I am sure you wish to state what is accurate—I ask you whether before she left Brighton, and before the fly left the door to take her to the railway to the lunatic asylum, you had not been informed that a certificate had been given by a medical man that she was not in a fit state to be removed? A. I don't know who informed me of it; I understood there had been such a certificate. — Q. Was not that before she left Brighton, before you got her into the fly? A. So I understood. — Q. Did you know to what lunatic asylum she was going to be taken? A. No, I did not. I did not know whether she was going to be taken to this Effra Hall, where she did go to, or whether she was going to the other asylum. — Q. Who gave the direction to take her to Effra Hall? A. I know nothing about all that. — Q. You knew she was going to be taken to a lunatic asylum? A. Yes. — Q. What necessity was there for taking her to a lunatic asylum at all? A. What necessity?

— Q. Yes, what necessity? A. If you will allow me to go into that. — Q. I will, you may go into that. A. In the first place, I considered she was unsound in mind, and a proper person to be in a lunatic asylum: in the second place, I considered, according to the information I had received, that she was living in a state of great discomfort, and that she would be, on the contrary, in a state of comfort. Do you want to know more? — Q. Who gave you that information? A. Besides that, I have every reason to believe there were people robbing her of her property, under the weak state of health she was in, and that she was not able to protect herself. — Q. Who gave you that information? A. Mr. Turner. You asked me why I thought she should go to a lunatic asylum, and those are my reasons. — Q. Who consulted you first upon this matter? A. Mr. Turner. — Q. Have you known him before at all? A. No. — Q. When you went into the room, did she appear to be alarmed at all? A. Not when I went in. — Q. I believe she screamed very much when the door was broken open? A. She might; I did not hear that. I was not near the door; I was in the drawing-room. — Q. It was broken open the second time when you went, was it not? A. Yes. — Q. You broke it open yourself, did you say? A. Did I say so? — Q. Who broke it open? A. Mr. Chase. — Q. Mr. Chase was the man with whom the grappling was—a man grappling with him? A. Yes, a very proper way of treating the representative of the Lord Chancellor. — Q. You do not mean to say that Mr. Chase represented the Lord Chancellor? A. Yes, he did. — Q. That little man (pointing to Mr. Turner), do you mean to say that he represented the Lord Chancellor? A. Certainly, as an officer of justice, of course he did. — Q. And represented him when the policeman took her away?—you all represented the Lord Chancellor? A. Yes; we should not have gone there on a fool's errand. — Q. Tell me what authority you had to take her away. A. I did not take her away. — Q. But you assisted? A. I stood by. — Q. You took a fly to see her off? A. Yes, to be sure I did; I wanted to see an end of the tragedy. — Q. The end of the tragedy which you laugh at? A. You make me laugh: you have got the order of mirth, you know. — Q. You wished to see the end of the tragedy, and this is the last act, I suppose? A. It is to be hoped so, for all parties.

Re-examined by Mr. Petersdorff.—Q. Now you have been asked some questions by my learned friend Mr. James, whether keeping one dog or one cat in a room is a strange thing, and you said, No; or driving one dog or one cat in a carriage, and you said, No. Now I ask you, would you call it a strange thing if a lady were to have three dozen cats in her carriage as her constant companions? A. Yes, I should. — Q. You have also been asked by my learned friend whether keeping five cats would indicate a state of unsoundness. Now supposing a lady who keeps five cats were to have a clean table-cloth laid for them and clean plates, and each of them a separate cup for milk, and were also to administer wine to them, and in addition to that, were to allow them to perform all the offices of nature in the same room in which she sleeps, and ate and drank, and never allowed the dirt they made to be cleared away, nor anything done to clean the room, what would you say to that? A. All these circumstances put together indicate unsoundness of mind. You must not go any further than that a person may be sound upon one point and unsound upon a dozen others. — Q. Now you have been asked about parents having had strong feelings of hatred against their children. Suppose an instance in which a daughter marries without the consent of her parents, and a year or two intervenes, and then they are perfectly reconciled, and live on happy terms together for many years; after that, the parent imbibes a feeling of hatred against the daughter without any apparent cause, would that, in your opinion, be insanity? A. Yes, I think so, undoubtedly.

Examined by the Commissioner.

Mr. Serjeant Wilkins.—There are one or two questions I would be obliged to you, Sir, to ask. Will you ask, first of all, whether it was not requested, particularly, by Mrs. Cumming herself, that either Mrs. Watson or Mrs. Hutchinson might go with her?

The Commissioner.—Q. Did she state, or express any wish, about any other person being allowed to go with her? A. I think she did; but I am not quite sure about that.

The Commissioner.—Q. Was the request made, and was it refused? A. That question makes me think there was a request made of that kind, and that the request was not acceded to.

Q. I think on the first day you signed what is commonly called a certificate? A. Yes; in the evening after the Commission. — Q. As a medical man? A. Yes. — Q. Was that a certificate that she was a person of unsound mind? A. It was. —

Q. Did you see any one else sign that but yourself? A. Yes, I saw Sir Alexander Morison sign it. — Q. Anybody else? A. And I think it was carried to Mrs. Ince afterwards. — Q. You have reason to believe that she signed it? A. I think she signed it afterwards. Q. You could give no authority for her removal, of course? A. Mrs. Ince may have signed it afterwards; only I and Sir A. Morison signed it at the time. — Q. She was removed away by the authority of Mrs. Ince, in fact? A. Yes. — Q. Did you see her off by the railway carriage? A. Yes. — Q. What carriage did she go by? A. First-class. — Q. Was she by herself in the carriage? A. No; the nurse and the nurse's brother, and one of the superintendents of police were with her. — Q. In the same carriage with her? A. Yes. — Q. They were by themselves? A. By themselves; I do not think any one got in afterwards. — Q. You were asked by the learned counsel, if it was any test of insanity that she was in the habit of taking cats in her carriage? A. I saw that it was a sort of a random thing of hers; I took it from her that she had two or three dozen cats in her carriage.

Mr. Serjeant Wilkins.—I would ask Dr. King whether, when she said she took two or three dozen cats out with her, whether she was not joking? A. She was not laughing; but she said, "O yes, two or three dozen."

*Dr. Monro*, called and sworn; examined by Mr. Petersdorff.—Q. I believe you are consulting physician to Bethlem Hospital and the Bridewell Hospital? A. Yes, I am physician to Bethlem Hospital, not consulting physician; I am senior physician to Bethlem Hospital. — Q. When did you first see Mrs. Cumming? A. I was to have gone to Brighton under the order of the Chancellor, but was prevented by illness. — Q. I will just draw your attention to the time when you actually saw her, and where you saw her? A. I saw her on the 6th and 8th of November, at Effra Hall, Mr. Elliott's asylum; and I saw her once more last Tuesday, on the 6th of January. — Q. Be kind enough to direct your attention to what passed between you and her when you saw her on the 6th of January? A. I found her in bed at Effra Hall, and we remained together something like an hour. — Q. Were you alone with her, or was any one present? A. The matron came up with me, and I think remained with me the greater part of the time; I am not quite clear of that; there was also a nurse standing near. Before I left, Mr. Turner came into the room, on the first occasion. — Q. Will you be good enough to tell us what passed, the inquiries you made of her, and what she said to you? A. I spoke to her of her daughters, and asked whether she had seen them. She exclaimed, "Don't mention them; they are the greatest enemies I have in the world; their only object is to rob and pilfer me of all that I have, and having obtained that object, I shall be free." She stated that Mrs. Ince, on one occasion, also attempted to strangle her. I questioned her further upon that, and she said she had every reason in the world to believe that that was her intention; she said that they had also attempted to poison her. The main topic of her conversation was strangling, and poisoning, and robbery, all of which she attributed to her daughters. I put some questions to her with respect to other points which I had heard of before, and she denied that she had ever accused Mrs. Ince of murdering her son. She also denied one or two other points of more importance; she told me she had never made a will; she could give no account of her property to me; I asked her the nature and amount of it—I could get no information at all. She stated that Mr. Haynes was her only friend in the world, and that Mr. Thorne was an enemy. She mentioned those facts which have been spoken of to me about the administration of milk to her, which Dr. Barnes had analyzed five years ago, and that then oxalic acid had been discovered; that it had been administered to a fowl, and the fowl had died. It is just possible there may have been by some chance oxalic acid in the milk, and Dr. Barnes may have analyzed it. I know nothing of that, but the main point, as it seems to me, was this, that she apprehended and feared that her daughters were going to poison her. I do not remember any other facts that passed; her mind was filled with apprehensions respecting her daughters robbing and poisoning her, and that was the main point which occupied her whole mind. I remained nearly an hour, and I left her on very good terms. She had only arrived a day or two from Brighton, I think. — Q. You say you saw her again? A. Yes, I saw her again on the 8th; she was then sitting up. Mr. Turner was not there on that occasion. I think the nurse was in the room, as far as I recollect; I stayed there not quite so long, perhaps forty minutes; I went over all the same points, which she confirmed in the same terms as she had stated them before, and I did not elicit any further facts. When I saw her on the 6th of January, last Tuesday, it was at her house, Gothic

Villa, Queen's-road, I then saw her in the presence of Mrs. Moore, and she received me in a very friendly way, and I had a good deal of conversation again upon the same points. I said, have you seen your daughter; surely you must have seen them? "Never mention them, never mention them; my bitter enemies." I asked her again about her property; I asked her whether she had 500*l.* a-year; she said, "Oh, no; nothing of the sort." I asked her whether she had 300*l.* a-year, she said, "Yes, it might be 300*l.* a-year." That was all the information I could get as to the nature and amount of her property. — Q. Did you ask her about what she had done with it? A. She said she had never made any will. She was quite confused as to any notion of the amount or value of the property; I gained, in fact, no information at all upon it, excepting what I told you about the 500*l.* and the 300*l.* — Q. From all the interviews and the opportunity which you had of observing her, did you form any opinion as to the state of the soundness of her mind? A. I certainly consider her of unsound mind; I certainly feel that this bitter feeling, with reference to her daughters, would influence her in every act of her life, and in the devising of her property. — Q. Could you form any opinion as to her capacity to manage her property? A. I have looked upon her as an imbecile; there is a good deal of playful shrewdness about her in some respects. She has powers of conversation upon many topics, but she, in my judgment, is an imbecile; she appears to me to be under the control of anybody that approaches her, as far as I can gather.

Cross examined by Mr. James.—Q. Are you aware, Dr. Monro, of the fact of the former commission? A. I am aware of the fact, because I remember, upon the occasion of the former commission, Mr. Ince called upon me, stating that he might require my professional services on that occasion, but it did not appear to be necessary afterwards, and I was not consulted. — Q. Were you aware, before you had this conversation with her, of the arrangement which was made, by which part of her property was given up to her daughters? A. I did hear it from Mr. Ince, or from Mr. Turner, I forget which, — Q. As to this feeling about her daughters, did you ask her the particulars, or the reason why she had that feeling; the expression you used was, that she said, "They were the greatest enemies; that their object was to ruin her, and to obtain all she had?" A. Yes; I asked her why. I think she said, they were disposed to ruin her, or do her personal violence in some way. — Q. Did she state, that by that arrangement they had obtained a considerable portion of her property? A. No; she did not. — Q. She did not allude to that? A. She did not. — Q. Now, as to this poisoning, did you make any inquiry to ascertain whether there was any foundation for that? A. I had no means of making any inquiries. — Q. Did you mention Dr. Barnes' name? A. Yes. — Q. In the report, you say that poison was given in a cup at that time by her daughters, which, upon being analyzed by Dr. Barnes, five years ago, was ascertained to contain oxalic acid, by which a fowl was killed. A. Yes; those were the words. — Q. She repeated that? A. Yes; on two or three occasions. — Q. This, perhaps is the accurate statement? A. Yes; I think those are the very words. — Q. You were very accurate in making the report to the Chancellor? A. Yes. — Q. You did not inquire to ascertain as to whether there was any foundation for that? A. No. — Q. You have made none at all? A. I may have mentioned it to Mr. Turner. I forget whether I have. I do not know Dr. Barnes; I never saw him that I know of. — Q. Did she mention one daughter more than another? A. She mentioned Mrs. Ince as the most hostile. She particularly specified that strangling; she attributed that to Mrs. Ince. — Q. Did she tell you the circumstance, that she entered the room suddenly, and put her arms round her neck? A. It was in the bed-room, and in her bed. I urged upon her the improbability of such an event, and she said, she had every reason in the world to believe it—the same expression she repeated last Tuesday. — Q. Do you remember her saying that she entered her room suddenly, and threw her arms round her neck? A. No, I do not recollect that; it appeared to me to be perfectly absurd. — Q. Did she not mention that fact, of her entering the room suddenly and throwing her arms round her neck? A. She stated that she put her arms round her neck; but I do not remember anything about suddenly. — Q. When did she say it happened? A. I forget in which of the villas it was; she removed several times; but I think it was at Maida-vale. — Q. You did not have much conversation with her about the cats? A. I did mention the cats, but I did not lay much stress upon the cats. If I am to speak of what I have heard in this place, I should say a good deal. — Q. But you said nothing to her about cats? A. I may have mentioned the subject of cats to her, but I did not push it to any extremity; I merely elicited that fact which was remarkable. — Q. Now, you say that upon that occasion she thought her income was about 300*l*. a-year? A. Yes. — Q. Did she tell you how it was derived? A. No. — Q. Did you ask her? A. I may have asked her. — Q. Did she tell you? A. I do not remember the source from which it was derived; I spoke of her houses to her. — Q. Did she speak of her houses to you? A. Not before I mentioned it to her. — Q. Did she not mention to you how her income was derived? A. No; I think not; but I will not be clear upon that point. — Q. You did not ask from what source her income was derived? A. No. — Q. How long did the conversation last? A. I should think about half an hour; it was up stairs in her bed-room, and Mrs. Moore was present. — Q. Is that the nurse who is with her now? A. I do not know that she is the nurse; she seemed more like a companion. — Q. I believe she is the wife of some physician, and a person who was named by the Chancellor to be present? A. I know nothing of her; I saw her once, and I have seen her since with Mrs. Cumming. — Q. She is the widow of a physician, and named by the Lord Chancellor, is she not? A. I do not know; but she seemed a very respectable person. — Q. She is the widow of a medical man, who was appointed by the Lord Chancellor to remain with her? A. Yes; she may have been.

*William Vesalius Pettigrew, Esq., M.D.*, examined by Mr. PETERSDORFF. — Q. You are the medical officer of Effra Hall asylum? A. Yes; I wish it to be particularly mentioned that I am a medical officer, not a proprietor; I wrote to the *Times*, it having been stated so, but they did not put my note in. — Q. You are the medical officer of that establishment? A. I am. — Q. How long have you been the medical officer of the asylum? A. I have been the medical officer of those patients that were at another asylum; previous to this it was all one asylum—a male and female asylum—for seven years, but now the female establishment is conducted at Effra Hall, and the other at Fulham. — Q. Still you have the experience of seven years? A. Seven years; there were forty patients at one house and twenty at the other. — Q. Do you remember Mrs. Catherine Cumming being brought to Effra Hall? A. I do. — Q. What day was it she was brought there? A. It was October the 30th, 1851. — Q. On her arrival, did you have an interview with her? A. I did. — Q. Did the interview between you and her take place when you were alone together, or were there other persons present? A. I think there were other persons present. The individual who brought her was present, and Mr. Elliott, the proprietor, who also, I believe, came from the station with her, he was there, and a sergeant of the police, I think, in plain clothes. — Q. Now will you state, as nearly as you can, what passed at the interview you had with Mrs. Cumming on her arrival? A. I said very little to her on her arrival; of course she was a little agitated; I happened to be there, it was a mere accident my being there. — Q. You say she was a little agitated? A. She was a little agitated; I asked the nurse who she was, and she stated that she was Mrs. Cumming, a relative of Mr. Ince's, and as I suppose, unfortunately, I avowed it at the time, as perhaps tending to increase the excitement, I said to her, "Oh I happen to know Mr. Ince very well," thinking to calm her by that as knowing some of her friends. — Q. What was the effect of that? A. She said directly, "Do not mention him! do not mention him! I have nothing to do with him." So I thought I had got into a scrape. Mr. Elliott stated that Mrs. Cumming was under the impression that her daughters and family had ill-treated her; upon which Mrs. Cumming said, "And so they have, and they want all my money; I will have nothing to do with them." I did not choose to enter into any conversation about that at that time, and I asked her if she was fatigued? she said yes, she was fatigued. I said, "Where do you come from?" She said, "From my own house." "Where is that house?" "In London, to be sure." I said, "Are you not aware you have come from Brighton?" "No, I have come from London." I then asked her if she would have some refreshment—if she would have a little wine. She said she should prefer a little brandy and water, which I immediately ordered for her, and left her. She was unexpected. — Q. Did you upon the average see Mrs. Cumming twice a week? A. Yes, more. — Q. Had you, then, on those occasions conversations with her from time to time? A. On each time. I never saw her without having conversation with her of various length. She went away about the end of November. I do not know the date. I had an opportunity of seeing her from time to time—ten to fifteen times, perhaps; scarcely so much as that—ten to twelve times, perhaps. — Q. I do not wish you to go through each interview. A. I could not go through each interview; it was all pretty much the same. On the first of November I made these few notes, and this is pretty well the conversation, at least these are pretty well the substance of the conversations at most times. — Q. Will you state to the jury the topics of the conversations? (The witness refers to a paper). A. This was two days after she came in; she said she knew she was in an asylum. — Q. This is the 1st of November? A. Yes, two days after her arrival. Knows she is in an asylum, and says she came here from her house in London. — Q. She repeated that on the 1st of November, did she? A. Yes; that was the reason I asked her again, to see whether the same impression still remained. Does not know where her house is situated in London. Came by the railroad, and had never come before by a railroad. She said her daughters were plotting against her. Some one endeavoured to poison her by drugged milk. On subsequent occasions, she repeatedly told me it was her daughters, and more frequently mentioned Mrs. Ince's name than anybody else's. I asked her how she knew the milk was drugged, and she said her cat refused to drink it; when it was put to her cat the cat refused to drink it. I asked her what made her give it to the cat; she said she had no particular reason, but the cat refused to drink it. I asked her what became of it; she said some one did it, at that time, but afterwards she said that Mrs. Ince had done it, and that her daughters had. — Q. First that some one, then Mrs. Ince, and then her daughters? A. Not at the same meeting. I may mention, that on subsequent occasions, on more occasions than one, I said to her, what makes you state that your daughters drugged that milk, had your daughters any communication with the milkman, or were they plotting with your servants against you? No, they had not, but she had done it, and it was done, and Mrs. Ince had done it, and it was poison. I asked her how she was certain the milk was drugged, and she stated that she had sent it to a chemist to be analyzed. I asked her what chemist? she did not know his name—but to the usual chemist, where she had her drugs. Subsequently to that, on two or three occasions, she told me she sent it to Dr. Barnes, who analyzed it. I asked her if she had no further proof that it was poison? She said yes, it was thrown into the fowl-house, and a chicken was poisoned by it. I asked her what poison it was? and on every occasion on asking her that, she told me that oxalic acid was found in it; and on two occasions she said that grains of arsenic were found in it. I mention this particularly, because she said grains of arsenic; but not on more than two occasions she said grains of arsenic; she always said oxalic acid; but on two occasions stated that grains of arsenic were found in it. — Q. Was anything said about tea, do you recollect? A. No, I do not recollect her saying anything about tea, it was milk; because I put the question to her then, that the milkman must be in collusion with Mrs. Ince; but she did not seem at all capable of reasoning on that or any such matter. — Q. Do you recollect any other conversation, I do not wish to lead you? A. Since that time, her cats had frequently refused to drink poisoned milk. I had heard of the statement about the cats, but I did not trouble much about that. I asked her if she was fond of cats? and she said yes, she was very fond of cats. I told her I was very fond of cats, too; however, I did not enter into much conversation about that.

The Commissioner.—Was that the expression, that they refused to drink milk, or poisoned milk?

Mr. Petersdorff.—Q. What was the expression as to milk? A. Frequently refused to drink poisoned milk, that was her expression. — Q. Anything else? She also stated, Mrs. Ince called to see her, and endeavoured to strangle her. I asked her where she lived at that time? and she stated at that time Mr. Ince was plotting against her liberty, and this occurred at Howley Villas. I know nothing about Howley Villas, but here is the expression she used. I asked her in what house it was? She said she was sitting at dinner, and her daughter came up and put one arm round her neck, and attempted to strangle her, but that she halloed out. — Q. Did you converse with her at all about her property. A. Very little. I asked her several times what property she had, and she did not know. When she came, I asked her if she had got any money in her pocket; no, she said, not a penny. I asked her if she knew anything about her property? No, she did not know anything about it; she had houses, but she did not know anything about them, or about their value. — Q. Did you ever talk to her at all about a will, or about making arrangements as to the disposition of her property on her death, or anything of that sort? A. No, I did not talk about that; I did not like to excite her, my habit is always to keep the patients as quiet as possible. — Q. With respect to her gesture, manner, and demeanour, was there anything remarkable? A. I have not the slightest doubt of it. The peculiarity in answering questions, the way in which she moved her arms about, and so forth.

Mr. Serjeant Wilkins.—Q. Not the slightest doubt about it? A. About her peculiar gestures, that I considered the peculiarity of gesture similar to that which persons of unsound mind use; her way of answering of questions—there was a peculiarity about it.

Mr. Petersdorff.—In speaking of her daughters, what was her manner, mild, or violent, or what? A. She was, when speaking of her daughters, generally excited; in fact, always excited to a certain extent. — Q. From the opportunity that you had of observing her and conversing with her, what is your opinion as to the state of her mind? A. I have no hesitation in saying, that she was decidedly of unsound mind. — Q. Have you formed any opinion as to her capabilities of managing property? A. No person labouring under such delusions, if they are delusions, with respect to her daughters, can possibly be able to manage her affairs, her money affairs, property affairs. — Q. What was the state of her bodily health during the time she was there? A. Her bodily health improved while she was there. — Q. Is she capable of moving about, walking about? A. She was capable of moving, she laboured under paralysis of the bladder, but the same night she came, the fire not being lighted in the room where she was, we asked if she would come down into the library, where the books are, it is called the library, and she walked down stairs with the assistance of a nurse. I am quite certain there must be twenty stairs, with a good wide staircase, but she walked down stairs, with the assistance of a nurse, and had her tea. — Q. Did she continue the whole time she was in the establishment walking and moving about? A. I never saw her moving about; when I went to visit her, she was either dressed and sitting on her bed, or else sitting in a chair near the fire.

Cross-examined by Mr. Serjeant Wilkins.—Q. If I understand you clearly, sir, you made known to Mrs. Cumming that you were a friend of Mrs. Ince's almost immediately on your introduction? A. I did. — Q. Were you aware at that time of her dislike to Mrs. Ince? A. About two years previously Mrs. Ince had asked me to go and see Mrs. Cumming. — Q. Will you be kind enough to answer my question? A. I must explain it. — Q. Answer the question, yes, or no. Were you at the time aware of the fact, that Mrs. Cumming entertained a strong dislike to the Inces? A. Not at that moment. — Q. Not at that moment? A. No. — Q. What do you mean by that? A. I mean this, that I had been aware there was some aversion two years previous, but at that moment I had forgotten the circumstance; I was perfectly unaware of Mrs. Cumming coming into the place. — Q. Have you attended Mrs. Ince yourself? A. I think I saw her once for a bilious attack, about a year and a half ago. — Q. Did you see her only once? A. As far as I am acquainted with that, only once for that bilious attack, but I have seen Mr. Ince several times. — Q. Have you any interest in this Effra Hall? A. Not a particle; I have less interest in it than any medical man had in any institution whatever. — Q. Do you think that violence and undue gesture indicates insanity? A. I do. — Q. Then be a little calm, please. A. I will; and hope I shall not have to retaliate. — Q. Were you connected with this establishment in the year 1847. Do you happen to know at that time the Commissioners intimated their intention to withdraw their licence from Effra Hall? A. I was not there at that time. — Q. Not in 1846. A. Not at any time that any intimation of the licence being taken away from that house had I been there, it is since that time that I was appointed. — Q. I thought you said that you were there seven years. A. About seven years; I do not know the time I did go, it was after that time. — Q. If you were seven years, I should think you would have been there on the 4th of February? A. It was in 1846 I went, but it was later than February, decidedly. — Q. Will you be kind enough, if you please, to give the gentlemen of the jury a definition of the word delusion. A. That which is stated and does not exist. Q. That which is stated and does not exist? A. As having occurred, and never did occur. — Q. Then you think if a person were to state something as having occurred which never did occur, that that would be, in your sense of the word, a delusion? A. Yes; with this addition, that when on being argued upon, and shown it could not have occurred, they still adhere to the same delusion. — Q. Suppose a person were superstitious? A. Then he is a person of unsound mind. — Q. Then you mean to say, that a superstitious person would be of unsound mind? A. Yes. — Q. Superstition argues an unsound mind, you say? A. In a certain degree. — Q. Then our forefathers were all mad? A. It may have been, there are very few who are not mad. — Q. Then at the time of the trial by ordeal, our forefathers were all insane? A. No doubt of it. — Q. Now I give you an opportunity of thinking, for your own reputation, think before you repeat that answer; you mean to say that superstition argues unsoundness of mind. A. Yes, I do. — Q. Then you believe that a man who believed in the trial by ordeal, which is called the ordeal touch, you believe that man was of unsound mind? A. Yes, I do; just as much as I should believe a man had not got his stomach right if he had a pain in it. It was not a healthy condition of mind, and it is not a healthy condition of stomach to have a pain in it. — Q. I think I can give you a definition, if I may be allowed to do so, without profaneness, which in my opinion is the best definition of a delusion, and I find the great Apostle of the Gentiles giving this definition of a delusion, and you will tell me whether you agree with it—"For they shall believe a lie." Do you believe that to be a good definition of a delusion? A. Yes; because they are made verily afterwards to comprehend it that it was a delusion. — Q. Who are made? A. Those to whom it was sent. — Q. I heard of incoherence. A. Perhaps, then, you will be kind enough to read your passage again, — Q. "For this cause God shall send them a strong delusion." A. What was your cause? — Q. No matter what the cause was, pray do be serious. A. I am serious. — Q. "For this cause God shall send them a strong delusion that they should believe a lie." I sought for some hours among different authors for a definition of the word "delusion," and that struck me as being the best I could find. Do you believe that to be a true definition of a delusion, "that they should believe a lie?" A. Yes, because it should be proved to be a lie afterwards. — Q. Because it would prove to be a lie afterwards? A. Yes, they should be cognizant that it was a lie. — Q. Is it a delusion to believe a lie? A. No, not a delusion to believe a lie. — Q. Is it a delusion to believe in the existence of that which has no ground? A. Yes, I should say it was? — Q. Supposing the foundation not to justify the superstructure, if I may use such a phrase, would that be a delusion? A. Well, I decline answering these questions in that way. I will not enter into what the definition of a delusion is. — Q. But indeed you must; you have come here to instruct these gentlemen upon this point. A. No; I do not come here to instruct you what a delusion, more than what insanity, is. I should like to see a man who could give a definition of insanity.

A Juryman.—You can give us your opinion, you know.

Mr. Serjeant Wilkins.—My questions are not only regular, but they are necessary.

A Juryman.—Quite so.

The Commissioner.—The questions are right enough, they are quite regular.

Mr. Serjeant Wilkins.—Q. Do you refuse to answer them? A. I refuse to enter into any argument on any scriptural affairs in this case. — Q. I do not want you to enter into any argument on scriptural affairs. I quoted from Scripture, and I said it without pretence, because I believed it to be the best definition I have ever seen. I quoted from Scripture the definition of a delusion, and I ask you, whether you think believing in a lie constitutes a delusion? A. If I consider believing in a lie constitutes a delusion? — Q. Yes? A. Believing in a lie to be a delusion? — Q. Yes, believing in that which does not exist, which is not true. A. If you know it to be a lie, it cannot be a delusion; he cannot believe in it if he knows it to be a lie. — Q. Then does believing in a lie constitute a delusion? A. No, certainly not, if he believes it to be a lie. — Q. How can he believe in it if he knows it to be a lie? A. If a man believes in a lie, and cannot prove it otherwise, until it is proved to be otherwise, you may consider it a delusion. — Q. I ask you if imperfect reasoning on pre-existing facts will constitute a delusion—imperfect or erroneous reasoning on existing facts, will that constitute a delusion? A. Will you repeat your question?

The Commissioner.—Q. Is imperfect reasoning on existing facts a delusion? No answer.

Mr. Serjeant Wilkins.—Q. Improper reasoning, or erroneous reasoning, on existing facts, is that a delusion? A. No. — Q. You know you stated just now, in your answer, which you gave us much more confidently than any other medical gentleman, we have had your opinion; you have said, there is no doubt but that she is decidedly of unsound mind? A. So I believe. — Q. Now, why? A. Because she has the same impressions on the same delusions, and it is continued whenever you speak to her about it. If the lady was here, I would show you in five minutes. — Q. You believe her to be insane, because she entertains delusions? A. Yes. — Q. Which is the delusion to which you refer? A. The poisoning of the milk by her daughters. — Q. In the first instance, she told you that somebody had put poison in the milk? A. Yes. — Q. Did she not tell you that Dr. Barnes and another gentleman had analyzed it? A. Yes. — Q. Did you take any pains to ascertain that that was true? A. Yes, I did. — Q. From Dr. Barnes? A. No, but from head quarters; she told me her daughters had done it, and I consider her daughters as good testimony as Dr. Barnes. — Q. You say she told you somebody had put poison in the milk? A. Yes; that was the 1st of November. — Q. She also told you Dr. Barnes had analyzed it? A. Yes. — Q. Did you go to Dr. Barnes? A. No; to her daughters. — Q. Was any person present at any of these conversations to which you refer? A. The nurse. — Q. When she mentioned the daughters? A. The nurse always. — Q. Is she here? A. I do not know; I have not seen her. — Q. What is her name? A. I do not know her name. — Q. Either of her names? A. I do not know either of her names. — Q. You know her when you see her? A. Perfectly. — Q. But you have not seen her here? A. No. — Q. Is not she the very same person that is now attending Mrs. Cumming at her own house? A. I believe she is. — Q. Did you regard her angry displeasure towards her children as a delusion? A. Yes; I said I thought it unnatural. — Q. Have you taken any pains to inquire into the history of their proceedings and dealings with their mother? A. Yes, I have; I took great pains about this case altogether. — Q. Were you aware that at one time during the mother's illness they never came near her for three years? A. No, I was not aware of that. — Q. Were you aware that they had confined her before in a lunatic asylum? A. Yes, I understand properly upon certificates. — Q. Were you aware she was taken away under the surveillance of a policeman, with a strait-jacket on? A. At what time? — Q. During the lifetime of their father? A. No, I was not aware of that; but she might have needed it. — Q. Were you aware of the fact? A. No; but she might have needed it. — Q. That may be, and she might not? A. She might not. — Q. Are you not aware, whatever be the real facts of the case, that she herself is under an impression yet that she did not need it? A. I suppose she is; I never knew an insane person that was not under the same impression. — Q. Were you aware that in 1846 they presented a petition for a commission? A. Yes. — Q. Were you aware that that extended over the period of eleven days? A. No; because I am not aware of the particulars of it. — Were you aware that there was no verdict, and that her daughters consented to the liberation, on a portion of the property being assigned to them? A. I was aware of that, through the *Times* newspaper, the other day, — Q. Did you not know it before? A. No. — Q. Were you aware, that in 1851, the very same persons presented a petition to deprive her of her liberty? A. This last November. — Q. Taking all these circumstances together, with her impression she is a sane person, do you think it unnatural she should entertain feelings of dislike and anger towards the persons who had caused all this? A. Taking all things into consideration, taking into consideration what I have read in the *Times* newspapers? — Q. No, do not; consider the facts I have laid before you; do you think it unnatural or unlikely she should entertain a strong feeling and dislike towards those persons? A. It depends upon whether she knows what the cause has been of it. — Q. I told you just now, you stated to me, in answer to a question from me, that she believed herself to be sane; under that impression, believing herself to be a person of sound mind, do you marvel that she should entertain a feeling of dislike towards the persons who sought to confine her, and allowed the jury to leave her at liberty on a division of the property? A. No; because many unsound persons have the utmost antipathy to their best friends. — Q. I ask if you mean to give that as a reason for their dislike? A. Of course, she would have a dislike to it; she is labouring under delusions altogether with regard to that. — Q. So you say; but I will show that she is not labouring under half the delusions that you are. I ask again, do you marvel at a person, tried as she has been, believing herself to be of sound mind, do you marvel that she entertains one reason? A. Believing her to entertain opinions that she is not insane, I do not marvel at it. — Q. What is your answer? A. Considering that she believes she is not insane I do not marvel at it. — Q. Should you think it unnatural that she should entertain feelings of dislike towards persons so treating her? A. Yes; because the circumstances could not take plaee. — Q. You might take my hypothesis entire. Supposing that that person always entertained a feeling of strong dislike towards the person so treating her? A. I think it impossible a person of sound mind could be so treated. — Q. You might take my hypothesis as I put it? A. I will allow your hypothesis, then. — Q. Supposing that true, should you wonder at a person of sound mind entertaining feelings of strong dislike towards a person irritating them? A. No; I will allow you your hypothesis. — Q. Did she not say, that when Mrs. Ince came up to the room, and put her arms round her neck, her daughters and her family at that time were plotting against her? A. Yes. — Q. Did she ever complain to you of the marriage of Mrs. Hooper? A. No.

Re-examined by Mr. PETERSDORFF.—Q. My learned friend asked you some questions with respect to the withdrawal of the licence of this establishment, do you know whether the parties who are now the proprietors of it, are the same parties? A. No; they are not the same parties.

Mr. Serjeant WILKINS.—Was he one? A. I believe he was, but it was on account of some family quarrels.

Mr. PETERSDORFF.—I understand you to say, these are not the same proprietors? A. No.

Mr. Serjeant WILKINS.—But Mr. Elliott was one of the proprietors? A. Yes; and there was a separation of partnership owing to some family quarrel, and the licence was restored; I was not the medical man at that time.

Mr. PETERSDORFF.—I asked you some questions of rather a medical character, but in what sense did you understand the word "superstition," when my learned friend used it? A. As a person superstitious. — Q. Superstition may exist with respect to a variety of facts. What construction did you put on that? A. A person believing in unnatural causes, defending a proposition on unnatural causes. — Q. When you speak of unnatural causes, do you mean physical failings, or mental theories, or what? A. I will give you an instance the Serjeant himself mentioned, by trial of touch. — Q. You would say that was superstition? A. Yes.

Mr. Serjeant WILKINS.—He said that was evidence of an unsound mind.

Mr. PETERSDORFF.—When you were answering questions as to what would constitute insanity, with respect to erroneous belief, will you explain what you think erroneous belief, so as to constitute insanity? A. A person who is impressed that a certain fact has taken place, or that something has taken place, and on arguing with them, and showing that it is utterly impossible that such can be the case, still he continues in the belief. — Q. I presume you would not apply the same doctrine to mere matters of theory, or doctrinal matters, on which differences of opinion might exist? Certainly not. — Q. Your answer applied to real demonstrable facts? A. Real demonstrable facts. — Q. Supposing the same dislike of the daughter had existed on the part of Mrs. Cumming prior to 1846, and before any attempt at all to prosecute the Commission, what would be your opinion then as to the state of her mind? A. I should say it was just the same.

*Hugh Welsh Diamond, M.D.*, sworn, examined by Sir F. THESIGER.—Q. I believe you have been for a great number of years accustomed to the care and treatment of insane persons? A. I have. — Q. For how many years? A. I should say for thirty years; as a child, my father had an asylum in which I was brought up, and I was his apprentice. — Q. I believe at the present you are a physician to the Surrey Lunatic Asylum? A. I am the resident medical officer on the female side. — Q. How many patients have you under your care? A. I have at this time 434; I have also an opportunity of visiting the males, amounting altogether to upwards of 800 patients. — Q. Your attention has been devoted for a great many years, has it not, to the consideration of the treatment of insane persons? A. It has. — Q. Were you requested to visit Mrs. Cumming in the course of last year, and did you see her? A. I was asked to see her one day in November, and I could not conveniently do so until the 16th. — Q. I believe you saw her in Effra Hall, in November, in the asylum? A. I saw her in November at Effra Hall. — Q. And in the following month of December, I believe you saw her at her own house, where she is now? A. I did. — Q. Now upon the first occasion of your seeing her, did you see her alone, or was there anybody in the room? A. There was a female servant; there were several on the second occasion. — Q. How long did your interview with her last upon that occasion? A. It was an hour to an hour and half. — Q. Had you been previously acquainted with the nature of what were called her delusions? A. Very imperfectly so. — Q. But so as to enable you to apply tests, if I may use the expression, to her mind? A. I might have learned more, but I thought I had enough to obtain any delusion or insanity if it did exist. — Q. Upon that occasion, did you find that she stated things which you considered were delusions? A. When I first went into her room, she declined to speak to me. She was eating her dinner. She had a breast of fowl, and was sitting at the table. I asked her to proceed with her dinner, while I warmed my hands a little. She then asked me what brought me there. I told her (I thought it better to be candid with her) that I wished to ascertain the state of her mind. She then seemed irritable, and said she had convinced a jury of her country already that she was of sound mind. I then said to her, I believe you have some daughters, Mrs.

Cumming. And she said, "Do not mention them." I said I had forgotten the name of the other daughter, but I recollected the name of Mrs. Ince, is she your daughter or your daughter-in-law? And she said, "You have spoken a true word; she is indeed a daughter-in-law, and not a real daughter." She told me her daughters were vile wretches (I think that was the very word or a similar word), and they had treated her very ill. I told her I had heard they had even gone as far as to attempt to poison her. And she said they had. I asked her to explain to me how that was accomplished. She told me Mrs. Ince had put some poison in her cream.

The COMMISSIONER.—Cream? A. Cream. I asked her how she had done it, and she did not reply. I said, how did you know poison was there?" She said, "I saw it." I said, had you any reason to suspect it? And she replied again, "I saw it." I asked her if it was curdled, that she saw it. She said, "No; it was not curdled." I said, how do you know it was poison then. And she said she sent it—I do not know whether she took it, because I do not know that she would walk, but my impression is, that she said she took it to a chemist, and that he had analysed it and pronounced it contained oxalic acid, and that he was right was proved by its having poisoned a fowl to which it was given. I asked, what did Mrs. Ince mean by putting this poison there? She said she could not answer that. I asked her whether she was always in the habit of taking cream, or whether this cream had been sent her as a present from Mrs. Ince. She told me she always took cream, and not milk. I asked her very closely whether the milkman had any hand in it at all; and she intimated that I could know something about it. She said, "Perhaps you know." I then said, it is a very serious thing for any one to attempt to poison you. She said, "Oh, they would do it again; at this time they would do it if they could." I said no more upon the subject of the poison, and I asked her how long she had been there. And she said, "Oh, a few days." I asked her where she came from, and she could not tell me. — Q. You say she could not tell you—what did she say? A. She looked confused, and could not reply at all. I said, do you know where you came from? And she did not reply a second time. — Q. You asked her where she came from, she looked confused and did not answer you? A. Yes, she did not answer me. — Q. What next? A. I said, you are a person of property; could you tell me anything of the nature of your property? She said, "I cannot answer you. You will answer, I am mad. I will answer a jury of my country," she said. — Q. Was there anything else? A. I do not remember anything else important. She said something about a will. I asked her, and she did not answer me distinctly. And she said, if she was only sure she had £150 a-year for her, she would be a happy woman—something to that effect—I know she said that 150*l.* a year would make her a happy woman.

Mr. Serjeant WILKINS.—Did I understand you to say a clear 150*l.*? I thought you said a clear 150*l.* a-year. A. No; I do not remember the word "clear."

Sir F. THESIGER.—You say this interview lasted about an hour and a half? A. From an hour to an hour and a half. I went in a fly, and kept the man waiting about that period. — Q. Did you find that it was easy for you to obtain these statements from her, or had you any difficulty? A. At first I thought she was irritated at my disturbing her from her dinner, but she afterwards was in a good humour and appeared agreeable, and wished me good day and so forth. I could have obtained any lengthened statement from her, but I was not aware of all this inquiry. I could have obtained any length of statement from her, and she would have given to me freely, but that fact of what I considered so palpable a delusion about seeing the poison, that I did not think it necessary to go further. — Q. What was the impression that that interview left upon your mind as to the state of Mrs. Cumming's mind? A. That she was a person of unsound mind. I would upon examination have signed a certificate that she was a person of unsound mind. — Q. And you entertained, of course, no doubt upon the subject? A. None whatever. — Q. Was there anything in her appearance or manner which struck you? A. Quite so. She was very much excited at a trifling thing, holding up her hands, and an uncertainty about her which you do not see, I think, in a person of sound mind. In speaking of her daughters the bitterness against them seemed to be great. — Q. You saw her afterwards at her present residence, Gothic Villa, I understand. A. Yes. — Q. What day was that? A. On the 29th. — Q. What persons were present at the time of that interview? A. I accompanied Dr. Davey.

— Q. And were there other persons in the room during that time? A. There was a lady whom I saw here during the first day of the inquiry with Mrs. Cumming, I think Mrs. Moore; and there were two or three other attendants standing about the room. — Q. How long were you with her upon that occasion? A. I should think about an hour, a short hour. — Q. Did she know you again at Effra Hall? A. She did. I said to her, you have seen me before, Mrs. Cumming. She said, "Yes, I did; I saw you at the madhouse." — Q. What did you say upon that occasion to her? A. I said very little to her. Dr. Davey spoke to her principally. I occasionally made some observations, but she declined. — Q. Do you recollect what passed with Dr. Davey and the lady? A. She declined to answer nearly everything which Dr. Davey put to her. She declined to answer, and then she became excited and would not restrain herself from answering relative to her daughters' ill-usage, and so forth, Mrs. Moore, who was present, said, "You are too weak, Mrs. Cumming, to answer, you will excite yourself," and so forth. And then Mrs. Cumming, in a low voice, said, "You are of the opposite party."

Mr. Serjeant Wilkins.—Who did she say that to—you? A. She said to me, —that was in reply to an observation I made, which was this, I said, "You seem, Mrs. Cumming, to be quite as well as when I saw you at Effra Hall, and then you answered me without any hesitation; and then she said, "You are of the opposite party." I also said, I have heard it stated that Dr. Conolly and Dr. Winslow had said you have partial paralysis of your legs; and she said, "They are too great gentlemen to tell a lie;" and she was very vehement when she made use of that expression, she held up her hand with great emphasis. — Q. As I understand Dr. Davey put various questions to her, did she say, I decline to answer, or was she silent? A. She declined to answer. — Q. Did she say, I decline to answer, or was she silent? A. She said, "I decline to answer," and of some she took no notice, but she frequently said, "I will answer that in court." Dr. Davey, I may say, was very frequent in his questions, and more than once, I may say, was very persevering in his questions, and more than once, I may say, Mrs. Moore said, "Dear Mrs. Cumming, you are very much fatigued, or in a very weak state."—Q. Did it appear to you or not, that Mrs. Moore was checking her from answering? A. It certainly did. — Q. Had you an opportunity of judging whether Mrs. Moore had appeared to have influence ever to prevent her answering? A. Undoubtedly she looked to Mrs. Moore; she cast her eyes to Mrs. Moore, and Mrs. Moore left off sowing, she looked up. — Q. Did she answer any questions? A. She did, with great excitement relative to the treatment of her daughters. — Q. Was that after what you stated? A. She could not restrain herself. — Q. And what did she say upon the subject of her daughters? A. I do not know that she entered into anything very particular, but she screwed her teeth together, and quite intimated they were unfriendly with her. — Q. She manifested her feeling against her daughter? A. Very much so. Dr. Davey said to her, "Have your daughters really ill-used you?" and she said, "Look to the papers, look to the courts, and there you will see it all detailed." — Q. Did she say anything more? A. I do not remember that she did anything that would impress me specially. — Q. Did it appear to you from that interview, from what you stated, that Mrs. Cumming was a person who was capable for a time of being controlled so as to prevent her delusions being shown? A. It did. I do not believe she could do it for any length of time; she would not be able for a dozen hours, or a less time than that. I should say if she was under free control for action a dozen hours in an asylum, anybody would obtain all the ideas from her. — Q. If she were left without any controlling influence for twelve hours, anybody even to have to see her, she would exhibit these delusions? A. I believe she could. I believe she could not be in one of my wards for twelve hours without anybody doubting her sanity. — Q. Do you consider that, from the interviews you have mentioned, you had a fair opportunity of judging of her state of mind? A. I do. — Q. And is it your opinion that she is or is not in a sound state of mind? A. I think she is in an unsound state of mind. — Q. Do you consider she is capable of governing herself and her property? A. I do not.

Cross-examined.—I am a Doctor of Medicine and Surgery, of Kiel, in Denmark. I am the only person in this country who possesses that degree, I believe. I think it a very honourable degree. I obtained it after writing a thesis on insanity. — Q. You have assumed, I presume, these impressions on her mind, with reference to the poison and with reference to the children, are delusions? A. I do. — Q. I do not think you will find it very difficult to give me the definition of the word "delusion." Will you give me your definition of a delusion? Such a delusion as constitutes mental unsoundness? A. A delusion is that where a person imagines a thing which really does not exist. — Q. But supposing a person under the stress of cirumstances to reason unsoundly or erroneously upon existing facts, you would not call that a delusion, would you? A. In this identical case she did not argue from facts. — Q. But answer that question if you please. Supposing a person,—because persons reason as their minds are disciplined, or according to their capacity, or according to their education,—supposing a person to reason erroneously on existing facts, that is, to draw unjust conclusions which a man of good reasoning powers would immediately discover to be a fallacy, would you call that such a delusion as to exhibit unsoundness of mind? A. If those facts are so far from the truth, I may say, or to ordinary belief they do not exist. — Q. For instance, supposing an untutored person on a dark night to have seen, for instance, a white horse in the distance, and to have brought his mind to believe that what he saw was an apparition, would you think it a proof of unsoundness of mind? A. Certainly not. — Q. Do you agree with the last witness, that a belief in the ordeal by touch would constitute unsoundness of mind? A. No, I do not. — Q. Supposing the lady to have been treated in the way that the papers to which she referred you would disclose—supposing her to have been taken from her house by two women and a police officer, in a strait-waistcoat—supposing her to have been confined for four months in a lunatic asylum—supposing afterwards a commission of lunacy to be taken out against her, and the jury to be discharged with the consent of the petitioner—supposing that during all that time, for ten days, she is subject to the gaze and questioning of various persons—supposing that time after time persons are sent to cross-examine her as to her state of mind—supposing that the detective police are sent in pursuit of her, and all these facts are brought to her knowledge, and she has ascertained this has been done by the sanction of her children, should you think her dislike of her children, under those circumstances a proof of insanity? A. Not alone, I do not. — Q. Now, supposing this—suppose that on a certain morning three of her fowls are found dead, and upon the same morning, at the bottom of a tea-cup, in some milk, she found something that looks very much like oxalic acid—that afterwards the contents of the crops of these fowls are analyzed by a physician, and are found to contain poison, and she came to the conclusion that what she saw in the cups on the same morning was oxalic acid, should you think that such a delusion as to constitute unsoundness of mind? A. I do not; but that is not the fact, as she represented it to me. — Q. I am taking this hypothesis—she said there was poison in the milk? A. She told me so, and told me she saw it. — Q. Supposing when the milk was turned out it was found there was Epsom salts at the bottom of that cup, do you think it would argue unsoundness of mind, three of her fowls having been poisoned that morning, if she thought Epsom salts was oxalic acid? A. I think with a weak-minded woman it would not be so; the way you state it to me is very different from the way she did. — Q. Would that argue unsoundness of mind? Supposing you should find, for several years one of her children had absented herself from her, and should rush into her room unexpectedly, and embrace her mother very tightly round her neck, the mother at that time believing her family were in league against her, and she came to the conclusion that her daughter was attempting to strangle her, and afterwards she was convinced she was wrong, would you think that would argue unsoundness of mind? A. No; that was an erroneous conclusion, certainly, if it was so.

Re-examined by Sir F. Thesiger. — Q. Her daughters had not been as attentive to her as daughters ought to be, and did she come to the conclusion that they were endeavouring to take away her life by poison, should you consider that that argued soundness of mind, or unsoundness? A. Unsoundness. — Q. Now supposing there may be a fact existing, but that fact to be considerably exaggerated in mind, and from a fact so exaggerated false conclusions to be drawn, should you consider that to indicate soundness or unsoundness of mind? A. I think in an extreme degree it would produce unsoundness of mind; and I think in this individual case the feeling is so strong against the children, so palpably strong, that it constitutes unsoundness of mind.

Examined by the Commissioner.—Q. You say you saw this lady in December last? Yes. — Q. There were other persons present? A. There were; there was Dr. Davey, Mrs Moore, and I think three attendants and women standing opposite to me; and I saw two standing behind the bed-curtain. There is a bed in the room, and two stood behind the bed-curtain, as if to hear what passed. There was a bed-curtain came along, and I saw two persons stand close behind the bed-curtain in the room. — Q. Mrs. Moore is a lady, I suppose? A. I do not know. I did not know her until I saw her in this room. — Q. Was she near Mrs. Cumming? A. She sat behind Mrs. Cumming—just behind her, if I may use such an expression. I should say with rather a Jewish cast of countenance. — Q. She was there in the room? A. Yes. — Do you think you had a fair opportunity, on that occasion, of judging of Mrs. Cumming? A. It would have been a very different thing. Yes, I am sure of it. She more than once said, "Dear Mrs. Cumming, you are very weak;" and then Mrs. Cumming immediately said, "You are of the opposite party." — Q. That was in answer to a question you put to her, that you had seen her at Effra Hall? A. No; that observation was at 59, Queen's-road? — Q. But I think you said, she said you had seen her before? A. Yes she did at the mad-house. She told me she had seen me before. — Q. Did you in any way remonstrate with Mrs. Moore? A. I did not. — Q. Did she know you were a medical man? A. She did. We were not allowed to see her until we respectfully sent our names in to her. — Q. She knew who you were? A. Yes; we were asked in a lower room; after waiting some five minutes, we went up stairs. — Q. And found her in her bedroom? A. With Mrs. Moore. — Q. It was after twelve o'clock in the day? A. It was about half-past one or a quarter to two. — Q. Mrs. Moore seemed to know who you were? A. Yes she did. — Q. You did not remonstrate with her? A. I did not. Dr. Davey was holding the main conversation I would say. — Q. He kept up the main conversation; but you asked her a few questions? A. Occasionally I put in a question. — Q. Did you attend her as a professional man? A. Yes. — Q. So that you did not have what you consider a fair opportunity of examining the lady. A. I certainly do. I consider it would have been a very different examination had she been left alone.

A Juryman.—In what sort of state was the room? A. A very clean and comfortable room, and so it was at Effra Hall.

The Commissioner.—You say Mrs. Moore was there, and three other persons? —two behind the curtain. Do you know the person we have talked of is the nurse from the asylum? A. No, I did not; I do not know any of them; I could not specify any of them, only Mrs. Moore. There were three other persons present. I could not tell who they were, and I should not know them if they were produced to me.

*James George Davey, M.D.*, sworn.—Q. You are a physician, I believe? A. Yes, I am. — Q. Are you resident medical-officer of the Middlesex County Lunatic Asylum? A. Resident physician of the Colney Hatch Lunatic Asylum. — Q. How long have you held that office? A. Since July last, the opening of the establishment. — Q. And I believe you were formerly one of the medical-officers at the Hanwell Lunatic Asylum? A. I was, some years, one of the assistant-physicians at Hanwell, under Dr. Conolly. — Q. I believe you have written a treatise on mental diseases? A. I have done so. — Q. I presume you have had great experience in such cases? A. For the last ten years of my life I have had very great experience in mental disorders. — Q. How many female patients have you under your care now? A. This moment I think I have 640, something like that—I will not be quite sure. — Q. Do you remember in December visiting Mrs. Catherine Cumming? A. I do. — Q. What day was that? A. It was yesterday fortnight. — Q. Was it upon the same occasion that Dr. Diamond has spoken of? A. It was. — Q. About what hour was it you went in the morning? A. It was between one and two, I think. — Q. In the afternoon? A. In the afternoon. — Q. Did Dr. Diamond remain in the room during the whole of the time that you were there? A. He did. — Q. Were any other persons there besides you and Dr. Diamond? A. There were three other persons present, a lady, I presumed, and two servants. — Q. You do not know the name of the lady? A. I have heard her name mentioned in this room. — Q. What was her name? A. I have heard it just now, but I did not pay much attention to it. — Q. Will you state, as nearly as possible, the conversation you had with Mrs. Cumming, and her manner and demeanour? A. On entering the room I saw Mrs. Cumming sitting on the right-hand side of the fire-place, looking enfeebled and in delicate health, with her lower extremities wrapped about with a blanket; and opposite to her sat a lady, and I took my seat on the left-hand side of that lady. I first of all said to Mrs. Cumming—I believe you are aware of the object of my visit, and I am a medical man; and she gave me to understand that she was quite aware of the object of my visit. I think I commenced my conversation by asking her if she had not lately been in the private asylum of the late Dr. Millengen, at Battersea. She hardly cared to give me an answer; but she did at length confess that she had been there in that establishment. I made some inquiries of Dr. Millengen, having some knowledge of him—some identical knowledge of him—and I asked her how long she had been there. She told me either five or two months, I forget which, but purposely I took the opportunity of putting the same question to her a second time. She made a different reply, on the second occasion, to that she did on the first. She told me, on one occasion, that she had been there five months; on the second occasion she told me she had been there two months. I immediately pointed out that discrepancy in her statement, and she seemed annoyed at my having done so, or rather seemed annoyed at her own imbecility of mind. I spoke to her also concerning Dr. Barnes. I asked her if Dr. Barnes had not been, or was, her attendant. She did not like to reply to that; she fixed her teeth firmly together, and looked angrily at me. I then asked if Dr. Barnes had not been required to analyse some fluid which she suspected to contain poison. She did not reply to me—she would not reply to me. To my repeated questions on various subjects, she said—"I shall not speak to you and shall not notice you; you came here intruding on me, and speak in an irritating manner; I shall make all my replies when I am in court." I also asked her if any other gentlemen had been to visit her—medical men. She would not reply to that question. I personally asked her if Dr. Winslow had not been to see her. She said he had; I think she said several times. I put some questions to her having reference to a conversation which Dr. Winslow may have had with her. She would not allow me to know what conversation had passed between that gentleman and herself, but merely said Dr. Winslow was a gentleman. I spoke to her also of her late husband, but she would give me no answer to any question I put to her on that head. I should say, throughout my interview with her, she treated me rudely, as no lady may be supposed to do, and gave me to understand that, if it were not for the common courtesies of life, I should be very soon shown the door. — Q. Were there any further topics to which you directed her attention? A. I did. I spoke to her about her daughters. I said I believed she had daughters. She did not reply to me then. Then I spoke to her again about her daughters. She was silent, and I said to her, at last, have your daughters been guilty, at any time, of acts of cruelty towards you? any acts of unkindness or cruelty, I think, were the words I employed; and she looked at me a moment, and I saw she was becoming influenced by what I said to her, and she was anticipating. She raised her left hand on high, and exclaimed, in a loud, sonorous voice, "Look at the papers and courts,"—I think were her words. It was either "look at the papers and courts," or "read the papers and courts." Throughout the interview she gave me abundant proof of being very imbecile in mind, and also of her being possessed with a variety of vague and unmeaning suspicions of everything and everybody. She was full of apprehension. — Q. Do you remember any particular statement she made with respect to suspicions? A. She had been so well tutored I thought that she was not allowed.

Mr. Sergeant Wilkins.—Really we cannot hear that.

Mr. Petersdorff.—You say there were three persons in the room besides you and Dr. Diamond? A. There were. — Q. Had you any opportunity of noticing whether any communication or influence was apparently exerted over her? A. Constant communications were being telegraphed from one side of the room to the other, and remarks made. — Q. From whom did these communications seem to come? A. More particularly from the lady on my right hand.

The Commissioner.—She was seated on one side of the fire? A. Mrs. Cumming was seated on the right-hand side of the fire-place, and the lady on the left-hand side of the fire-place, and I was sitting on the lady's left hand; Mrs. Cumming was opposite to me. — Q. You were opposite Mrs. Cumming, and the lady was opposite Mrs. Cumming? A. Exactly.

Mr. Petersdorff.—You said you had a difficulty in obtaining an answer from her to the questions you had put? A. I had. — Q. Did anything occur to enable you to say why you did not obtain these answers? A. I considered, from the influence which was exercised upon the mind of Mrs. Cumming by those present. — Q. Did you talk to her at all about her property? A. I did not—I think I did not. — Q. Or about the will? A. No, I did not. — Q. Was there anything else that you remember passed at this interview? A. I do not remember anything else. — Q. From what you heard do you think you had an opportunity of forming an opinion as to the state of her mind? A. I conceive that I had an abundant opportunity of forming an opinion. — Q. And what is the result? A. My opinion is that she is decidedly of unsound mind. — Q. Could you form an opinion as to whether you think her capable of managing her property and her own affairs? A. I am perfectly convinced that her imbecility of mind must prevent her from managing any affairs of any kind. — Q. I believe you did not call again? A. I did not.

Cross-examined by Mr. Serjeant Wilkins.—Q. Are you a contributor to a work called the "*Zoist?*" A. I am. — Q. Do you believe in mesmerism? A. Most certainly I do, and so do all right-thinking men. — Q. Then every man who does not agree with the notion of mesmerism, is not a right-thinking man? A. He either does not think sufficiently, or he is very prejudiced. — Q. Do you believe in clairvoyance. A. I do. — Q. Do all right-thinking men believe in that? A. All those who have investigated the question; but I do not see what relation that has to the question at issue. — Q. You do believe in clairvoyance? Pray have you stated that you have cured insane persons by the influence of mesmerism? A. I have cured three persons by mesmerism. — Q. What is your definition of a delusion? A. A belief in that which does not exist. — Q. You think that is a perfect definition of a delusion? A. No, I do not pretend to give a definition of it—in a general sense perhaps. — Q. For instance, I am not a right-thinking man, for I do not believe in the existence of clairvoyance? A. Because you have not had sufficient opportunities. — Q. I beg your pardon. Never mind whether I have had sufficient opportunities; I do not at all object to break a lance with you, physician as you are; but do you say that the fact of my assertion that clairvoyance is nonsense, argues unsoundness of mind on my part? A. To a certain degree. — Q. I am very glad I got that answer—it is what I anticipated. Then you would say, every gentleman in the room who asserts the same thing, is, to a certain degree, unsound in mind? A. His mental state is to be pitied; he does not know what is true. — Q. How the gentlemen of the jury will eat their lunch after that I do not know. Allow me to ask this, does the "consciousness of one's imbecility or weakness of mind" argue insanity? A. Yes. — Q. Suppose, for instance, I were bowed down with my consciousness of my own ignorance and my own unsoundness of mind, would that consciousness argue insanity? A. Many insane persons are conscious of their insane indications. — Q. That is not an answer to my question. Does the consciousness of weakness of mind argue insanity? A. Why it may or it may not; it would depend on circumstances. — Q. Would you not rather say, that the wisest men are those who generally smart the most under the consciousness of their own incapability? A. They are wise men who do so. — Q. What do you term imbecility? A. Intellectual incapacity. — Q. Intellectual incapacity? A. A feebleness of intellectual powers. — Q. Feebleness of intellectual power is imbecility—what extent of feebleness? A. That would depend upon age, sex, and other circumstances. — Q. Feebleness of mind in a person of the age of seventy, is that imbecility? A. It may or it may not be, according to collateral circumstances. — Q. Then will you give me such a definition as I can make use of and rely on? A. It would depend upon the temperament of the individual, and the previous history of the individual, and the state of his general health. — Q. Well? A. Every case must stand on its own merits, I take it. — Q. I do not know that exactly; there are some cases that may be tested by general rules. Now I want to know, for the information of the jury, what you mean by imbecility? A. A feebleness of mental power. — Q. Will you be kind enough, if you please, to explain to the learned Commissioner and the jury, what you mean by persons telegraphing in the room? A. Why I meant by that, that the lady sitting on my right-hand looked more to Mrs. Cumming than she need have done, had she not been anxious to restrain her from the expression of feelings which may have gone far to prove her insane. — Q. Is that what you mean by telegraphing? A. That is what I mean by telegraphing—looking frequently towards her and at her. — Q. As you can interpret looks so well, did not

Mrs. Cumming several times look most beseechingly to that lady? A. I saw her look frequently towards that lady. — Q. But you interpret her looks. I ask you if Mrs. Cumming did not look beseechingly to that lady? A. She looked at her frequently, and it appeared to me she looked at her lest she should commit herself. — Q. I ask you if she did not look beseechingly at that lady? A. I am not aware that she did. — Q. Did you press her? A. Yes, I admit that I did. — Q. I should presume, then, that if you saw any interruption, knowing the authority with which you were clothed, I should presume that you reproved those parties whom you suspected of telegraphing? A. Most certainly I did not, for the best of all reasons— I wished to understand exactly the position in which they were placed towards each other. I wished to understand what it all meant. — Q. What what meant? A. This telegraphing. I wished to find out the real state and condition of this lady. — Q. Can you give any other instance of that which you call telegraphing, beyond that you describe? A. No. — Q. Your answer to my learned friend was, that they were telegraphing about the room—I have not taken down the exact answer? A. I think it was across the room. — Q. How many persons telegraphed? A. The lady on my right-hand I saw particularly engaged in it during the whole of my interview. — Q. What is that? A. This telegraphing. — Q. Looking, is it? A. Looking in a peculiar and significant manner from time to time. — Q. Did you complain at all before you left the room of any telegraphing? A. No, certainly not—I had no reason to do so. I did not wish to do so.

Re-examined by Sir F. Thesiger.—Q. You say you made no remark with regard to what you call the telegraphing? A. No, I did not. — Q. You have given us the reason, that you wished to see what was the position of all parties? A. Certainly. — Q. I suppose your object partly was to see whether she would be influenced by those who were about her? A. That was exactly my object, to observe the strength of her volition. — Q. Now, you have been asked for various definitions by my learned friend, is it your judgment that there can be any general definition given of insanity or unsoundness of mind? A. I think there cannot.— Q. From your experience, are you able to say whether you can apply general rules unerringly, or whether each case must not depend on its own facts and circumstances? A. I think each case must depend on its own facts and circumstances. — Q. You have been asked particularly, with regard to imbecility, what is your definition of imbecility. Would you ascribe the appearance which you have represented the state of Mrs. Cumming's mind, would you ascribe that to imbecility, the sense in which you have given your definitions? A. Most certainly. — Q. Or to old age? A. Decidedly not to old age; it was an imbecility, a disease, which I saw in Mrs. Cumming.—Q. I will not enter particularly into the question of mesmerism and clairvoyance, and tell you how far I believe or disbelieve in it; but I believe there are persons of very considerable eminence who believe in clairvoyance and mesmerism? A. Of the highest eminence in all parts of Europe and America.

Examined by the Commissioner.—Q. Did they understand in the room that you were a medical man? A. They did. I made it a point of mentioning the object of my visit when first I entered the apartment.

A Juryman.—You did not consider that you possessed power to order them out of the room? A. I did not wish it. — Q. Did you possess the power? A. I am not aware that I did.

Mr. Serjeant Wilkins.—Certainly.

Witness.—I did not wish to do it.

A Juryman.—Were you aware you possessed the power? — A. I was not aware of it.

Mr. Serjeant Wilkins.—Is this asylum of which you speak at Colney Hatch? A. Yes. — Q. I believe it is for incurable patients, is it not? A. No, it is the sister asylum to Hanwell.

*Charles James Berridge Aldis*, Esq., M.D., sworn, examined by Sir F. Thesiger. —Q. You are a physician, I believe? A. I am. — Q. And a fellow of the College? A. I am. — Q. And are you a lecturer on medicine at the Hunterian School? A. I am. — Q. I believe you have not devoted yourself exclusively to cases of insanity? A. No. — Q. But in your general practice they have come within your knowledge? A. They have. — Q. Have you had considerable practice in those cases? A. I have had a fair share of experience. — Q. Were you requested to visit Mrs. Cumming, and did you do so, and when? A. I was requested to do so, and I visited her at Effra Hall on the 16th and 17th of November. I visited her again on two occasions at Gothic Villa, St. John's Wood, on the 28th of November and on the 3rd of January.—Q. Upon the first occasion of your visiting her at Effra Hall, how long did you remain with her? A. About three quarters of an hour. — Q. Were you alone with her, or was anybody else in the room? A. There was a nurse in the room. — Q. One of the nurses of the establishment? A. One of the nurses of the establishment, and I think the matron was present. I heard a person near the door, which led into another room, and I asked the matron afterwards if she had been in the room. She said she heard what I stated, so I concluded it was the matron and one of the nurses. — Q. I believe your first visit to her was on a Sunday? A. It was. I had appointed Monday, but having received a letter from Mr. Turner, containing an urgent request that I should visit her, I was compelled to visit her on the Sunday. — Q. Did you make an apology for coming before she was up? A. I did. I said I regretted coming so early, before she was up, and I said that I regretted that it was likely to disturb her. She said, "Oh! you do not disturb me; no one has disturbed me since I have been here, for I have seen no one." — Q. Did she say she had seen no one, or that she had seen nobody she expected? A. Except through the window, she had seen no one, she had not been disturbed since she had been there. — Q. Therefore, she was not irritated or excited by your visit? A. Not at all; she seemed quite composed, and gave that answer in a cool, quiet manner. — Q. Did you enter into a conversation with her? A. Before entering into a conversation, she became suddenly very much excited. — Q. How long after you came into the room was that? A. Very soon after she made this remark, very soon. — Q. How did she show her state of excitement? A. There was a closing of the teeth, and subsequently a gnashing of her teeth; her teeth closed together, and there was an excitement, and a degree of irritation showing itself, and then she became incoherent. — Q. Now, will you be kind enough to explain what you mean by that? A. She rambled; she spoke in a rambling manner. If you will permit me to remark, she spoke so quickly and rapidly that it was impossible for me to retain all she did say, it was so remarkably quick; but I will tell you what I was able to remember. — Q. Could you at all, though she spoke in this rapid manner, collect the subject on which her mind was bent? A. Yes, I collected something. She commenced something about the railroad, that she had been dragged along the railroad, that she had never been on the railroad before. She then spoke of the asylum, the persons she had seen at the asylum. She then spoke with great bitterness of her daughters, and particularly of her daughter, Mrs. Ince, who had been drinking at the Horns Tavern; that she was very much disgusted with her; that she had brought her up differently. She spoke this in a most rapid manner, and I viewed it to be rambling and incoherent; and, in fact, it was quite so, for there were many things that I could not recollect; it was verily an incoherent mode of talking. — Q. I observe you mention that she stated first she had seen nobody at all, except through the window, while she was there, and then you once said she spoke of the persons who had visited her? A. She spoke of persons whom she had seen in the asylum.—Q. You say she spoke of her daughter, with bitterness, I think your expression was? A. Yes; but particularly of her daughter, Mrs. Ince. — Q. Will you be kind enough to tell me, when she spoke in this way of her daughter, whether you asked the cause of her feelings against her? A. Yes; she was calmer after a time, and I then pressed this point as to her having seen her daughter drinking at the bar of the Horns Tavern. I asked her if she was positive of it. She said she was quite positive of it; she had seen her herself; and this appeared to be the only cause, in fact, that she assigned to me for such a great dislike to Mrs. Ince; that was the only cause I could ascertain from her on this occasion. — Q. Did she give, on any occasion, any reason for her dislike of her other daughter, for you say she expressed bitterness against both her daughters? A. Generally speaking, when her daughters' names were mentioned, she spoke of them; but I do not recollect any particular expression as regarded Mrs. Hooper. As far as I can recollect, it was against Mrs. Ince. — Q. Did you press her upon the subject of the reason of her feeling against Mrs. Ince? A. I spoke then about it, and she said she had seen her, she was certain about it. — Q. And there was no other cause assigned? A. There was no other cause assigned. I asked her if she had attempted to strangle her, or to poison her, but she made no reply. I think she said she would tell me elsewhere all—tell somebody elsewhere, in another place—but she gave no reply to my question I put as to her having been strangled or poisoned, or an attempt having been made.—Q. Did you put any question to her about her property? A. I asked her the amount of her property, and she would give me no reply. She said it was fast running away. She did not tell me the amount of her property. Q. Did you ask her anything about a will? A. I asked her if she had made a will, and she said she had not; subsequently, she said she had. — Q. In the same interview? A. In the same interview; and then I asked her to whom she had bequeathed her property, but she did not reply. — Q. When she told you she had made a will, did she say whether that had been made recently or at any distant period? A. She did not make any remark: a long time ago, I think she said. I think she had made a will a long time ago—if I recollect rightly—a long time ago: she did not mention the period. — Q. Did you inquire of her whether she had any friends who would protect her, and take care of her? A. I think she said she had no friends who would take care of her. — Q. I believe that she was comfortably circumstanced at Effra Hall, was she not? A. Quite so. — Q. It is a very nice place, quite like a country residence, is it not? A. Quite so; like a private gentleman's residence—no high walls; there is an open railing in the front of it. It had the appearance of a gentleman's house. — Q. Upon the first occasion you saw her, were you able to ascertain the state of her mind, and what was your opinion of her? A. It was my conviction that she was of unsound mind. — Q. Upon the second occasion, you saw her at Effra Hall? A. I saw her in the afternoon of the next day. — Q. Did you examine her, to see in what state she was as to her bodily health? A. I did; I examined her pulse and her tongue; her tongue was clean, her pulse was regular, and she seemed in a tolerably good state of health—tolerably good—but I took into consideration that she had had paralysis of the bladder. — Q. Did you converse with her on that occasion? A. I did; she was composed, in fact composed during the whole of that visit. She was sitting up then—it was the afternoon; she was sitting up by the fireside, but she still persisted in saying that not only Mrs. Ince had been drinking at the bar of the Horns tavern, but that Mrs. Hooper had been doing the same thing. — Q. Did she say anything about her daughters, as to any food, or anything of that sort? A. There was a small quantity of cold roast beef upon the table, and she pushed it towards me, and said, I can get nothing else for dinner but this bone in the asylum—in this place—and I then observed, that no doubt her daughters, if she required any delicacies, would send her some; and she said, Oh, I should be afraid to touch anything that came from them—afraid to touch it. She also said she would not condescend to touch it; she also said that. — Q. Did you make any inquiry of her, upon that occasion, as to her property? A. I asked her the amount of her property, and she could not tell me anything about it. — Q. What did she say? A. She did not seem to know anything about it at all, further than I think she did say, upon this occasion, that a Mr. Ebenezer Jones had made a sale of some property; that he had sold something, but that she would not have it done—as if it had been done, and was rescinded afterwards—as a matter of course, it was a kind of way of expressing herself, but I think that she made that remark. — Q. Did she say anything about Mr. Haynes upon that occasion? A. She said that she had been told that she had only one friend; she had been told that Mr. Haynes was her only friend. — Q. Did you ask her whether she had seen anybody; do you recollect? A. Oh yes, I do perfectly, because I was particularly anxious about it; to test her memory, I asked her if she had seen anybody, and she still persisted in saying that she had seen nobody since she had been at Effra Hall, although her medical attendant, Dr. Caldwell, had not long left. — Q. Now your third visit, I think, was to go to Gothic Villa? A. It was. — Q. When you went there, you found her in bed, I think? A. I did.

The Commissioner.—When was that? A. The 28th of November.

Sir F. Thesiger.—On going in, what did you observe? A. On going in, I was accompanied by Mr. Turner, and I found she had the curtains closed, excepting on one side; on approaching the side where the curtains were closed, on opening them to make an examination of her, she, on attempting to open them, exclaimed, very loudly, "Don't do that; I cannot bear it." Well, wishing to ascertain what this meant, I waited, for a single moment, and went to the other side of the bed, where the curtains were open, and I immediately discovered that the right eye was very much injected, that there was great intolerance of light; it was very red, and she suffered from great pain I presume in the eye, for there was great intolerance of light; she could not bear the least light, and this was the cause of her exclamation; and I removed the cause as soon as possible, to occasion as little inconvenience as possible, and then asked her respecting the persons she had seen at Effra Hall; she said she had seen nobody excepting Mr. Haynes. I asked her where she resided in 1847 and 1848? she could not tell me. — Q. You say she could not tell you, or would not tell you? A. She could not; she did nôt seem to be able to understand it at all; she did not refuse to answer the question. — Q. But did not seem to be able to tell you? A. But did not seem to be able to tell me: when I then asked her about her property, she again said it was fast running away; and, on making that remark, Mrs. Moore interrupted her, and said, I think it will be better not to excite her. Though I thought Mrs. Cumming could bear a longer examination, I yielded to Mrs. Moore's request, and did not wish to be thought to excite her unnecessarily, and I discontinued my examination—my examination was a very short one, but I was merely testing her memory. — Q. Was any one there besides Mr. Turner? A. Mrs. Moore and Mr. Haynes. — Q. You did not mention Mr. Haynes? A. I did not. — Q. Did you find him there when you went in? A. We found him there when we went. — Q. In the room? A. Yes; and Mrs. Moore was there—he was there at the time I made the examination. — Q. Do you remember Mr. Turner and Mr. Haynes leaving the room, and Mrs. Cumming was going on talking? A. Yes; and I think I walked to the other end of the room, and looked out of window; and, not wishing to be thought to excite her; but, as soon as Mr. Haynes had left the room, Mrs. Cumming would not cease; she went on in a rambling manner, and the curtains were closed, and I could scarcely hear what she said; but she went on rambling, and Mrs. Moore kept on looking towards her, and doing so—" Sh" " sh."— Q. Did that appear to have any influence? A. No, she was talking and muttering to herself still. — Q. I believe you saw her once again, did you not? A. I saw her once again on the 5th of January, she then declined to answer any questions that I put to her. — Q. Who was present then? A. Mr. Turner and Mrs. Moore. — Q. But she declined to answer any questions? A. Yes, declined to answer any questions; I think she said she would answer in another place. — Q. In your opinion, was Mrs. Cumming of sound mind? A. In my opinion, she is of unsound mind. — Q. In your judgment, is she capable of managing her affairs and herself? A. In my judgment, she is not.

Cross examined by Mr. Serjeant Wilkins. — Q. Are you a physician? A. I am a physician. — Q. Do you practise otherwise than as a physician? A. No, I do not. — Q. Your main practice is, I believe, amongst sane people? A. Principally. — Q. Did you ever have anything to do with an asylum? A. No; I attended at Battersea. — Q. A patient of your own? A. No, merely to become acquainted with the disease as much as possible. I attended Sir Alexander's practice some little time, and also his lectures; and also in various lunatic asylums. I read as much as possible to make myself acquainted with the disease. — Q. Are you on intimate terms with Mr. Ince? A. I have now been off and on for about twenty years. I have been to his house, but not as a visitor. And his son has been at my house; and I met him twenty years ago in the board-room of St. George's Hospital. I know him to speak to him very well, but we have not visited. — Q. Now, when you went to this lady's room, at her residence, you say her right eye was much injected; by that you mean, I suppose, in plain English, very much "bloodshot." A. Very much bloodshot. — Q. And you found there was a good reason why the curtains should be drawn—because the light pained her. A. Quite so. — Q. Do you know the lady who is attending her is the widow of a physician, Mrs. Moore? A. I did not know it. Q. Did she not tell you Mrs. Cumming had had so many medical men with her, and that she had had a troublesome night; she could not bear any excitement. A. She begged her not to excite herself. — Q. Did she not tell you she had had medical men with her that morning; and several days before that she had had a very uneasy night, and begged she might not be excited? A. I do not recollect those words. — Q. Or to that effect? A. I do not recollect anything about a medical man. I immediately gave over. — Q. When Mrs. Moore said "Sh! Sh!" did it not appear to you an ordinary precaution an ordinary nurse would use to a lady in a state of excitement? A. I do not know exactly in what way to take it. — Q. What induced you to come to the conclusion she was of unsound mind? A. Incoherence at the first visit. — Q. Explain what you mean by incoherence? A. Rambling. — Q. Were not every one of those subjects of grievances of which she complained? Did she not begin by complaining of having been dragged on the railroad, never having been on one before in her life? A. Yes. — Q. Did she not complain that she could see no one, but through the window? A. Yes; she had seen no one. — Q. Did she not complain that she could see no one but through the window? A. No; she had not been disturbed; for she had not seen anybody, except through a window. — Q. Did you ask her if she had seen any of her friends? A. Yes. — Q. Friends? A. Yes. — Q. Did she not, in answer to that say, "why, how can I see any of my friends, when I can see no one except through the window?" A. Yes, she made a remark of that kind. — Q. And when at this cottage, she said, no one had called on her but Mr. Haynes; was not that just after she told you she had no friends to take care of her; and did she not say Mr. Haynes was the only friend who had called upon her at the madhouse? A. Yes; she said she had seen nobody except Mr. Haynes. — Q. Did she not say he was the only friend who called upon her? A. No; I remember now; it was suggested to me by the matron, that perhaps she does not consider all the persons that visit her as friends; and I changed the word on several occasions, and put anybody, or any person. At Gothic Villa particularly, I tried her in different ways. I changed the word, I remember now. — Q. Were not those incoherences of which you speak, a rapid succession of complaints on her part; a different notion of the sufferings she had undergone, and was undergoing? A. To a great extent they were; but I could not connect the bar of the Horns Tavern with that incoherence. — Q. But that was an answer to a question? A. No; she rambled in that way. — Q. You said you asked why she had disliked the daughter; and she said the only reason was because she had seen her drinking at the Horns Tavern? A. No, that was not so. — Q. Did that form one of her complaints? A. Yes. — Q. Did it appear singular to you, that a person who had suffered a series of ills, should repeatedly repeat them, or recount them—or should be constantly dwelling upon them? A. No, it is not remarkable. — Q. Was there anything besides the incoherences, that led you to suppose her unsound? A. She had a dread of tasting anything that came from her daughter. — Q. That dread was expressed in those words; you said, the daughters, if she wished it, would send her some delicacies; and her answer was, "I should be afraid to eat after them; and would not condescend to receive anything at their hands?" — A. Yes; I should be afraid to touch anything that came from them, after what she said. — Q. And "I would not condescend to receive anything at their hands?" A. Yes. — Q. Is there anything else? A. No.

Sir FREDERICK THESIGER.—I understand that all the wine and other things that came to Effra Hall, came from her daughters?

Mr. Serjeant WILKINS.—She did not know it, I suppose. Do not for a moment suppose that I mean to insinuate that her daughters would do anything of the sort.

Sir FREDERICK THESIGER.—But any delicacies she had, such as wine, or other things, were all sent by the daughters.

Mr. Serjeant WILKINS.—You may take that for granted, Sir Frederick.

(This closed the case of the Petitioners.)

